# Energy metabolism and adaptation to hypoxia in the non-photosynthetic green alga *Leontynka*

**DOI:** 10.1186/s12915-026-02529-3

**Published:** 2026-01-29

**Authors:** Pia Corre, Jana Pilátová, Tomáš Bílý, Eliška Zadrobílková, Ivan Čepička, Marie Vancová, Martin Lohr, Oliver D. Caspari, Marek Eliáš, Tomáš Pánek

**Affiliations:** 1 Department of Zoology, Faculty of Science, Charles University, Prague, Czech Republic; 2 Institute of Physics, Faculty of Mathematics and Physics, Charles University, Prague, Czech Republic; 3 Lawrence Berkeley National Laboratory, Molecular Foundry, Oakland, CA USA; 4 Institute of Parasitology, Biology Center, Czech Academy of Science, Budweis, Czech Republic; 5 Laboratory of Electron Microscopy, Biology Center and Faculty of Science, University of South Bohemia in České Budějovice, Budweis, Czech Republic; 6 Institute of Molecular Physiology, Johannes Gutenberg-University, Mainz, Germany; 7 Institute of Microbiology and Biotechnology, University of Bonn, Bonn, Germany; 8 Department of Biology and Ecology, Faculty of Science, University of Ostrava, Ostrava, Czech Republic; 9 Present Address: Center for Epidemiology and Microbiology, National Institute of Public Health, Prague, Czech Republic

**Keywords:** Chlamydomonadales, *Leontynka pallida*, *Leontynka elongata*, Energy metabolism, Anaerobiosis, Evolution, Leucoplast, Mitochondrion, Cristae, Carotenoids

## Abstract

**Background:**

*Leontynka* is a non-photosynthetic lineage of the order Chlamydomonadales (Chlorophyta). Although many Chlamydomonadales members encode components of the anaerobic energy metabolism, studies focused on Chlamydomonadales algae thriving in hypoxia and not prospering in oxic conditions are missing. Using a combination of experimental approaches, comparative genomics, and advanced in silico protein localization analyses, we employed *Leontynka* as a model to investigate the evolution of anaerobiosis in Chlamydomonadales.

**Results:**

*Leontynka* spp. accumulate a wide range of storage forms, enabling them to cope with nutritional stresses. Their mitochondria contain well-developed cristae mediating a conventional aerobic energy metabolism. Moreover, colocalization of a Raman signal for cytochromes with the position of mitochondria in the cell indicates that oxidative phosphorylation is an important route of energy metabolism in the alga. Interestingly, *Leontynka* spp. concentrate enzymes potentially involved in oxygen-independent ATP synthesis within the plastid, which lost the ability to produce ATP using proton gradient generated by membrane complexes that exploit redox reactions. We analyzed the composition of prokaryotic communities co-isolated with *Leontynka* spp. and hypothesize that their preference for hypoxic/microoxic conditions is facilitated by metabolic interactions with certain microaerophilic and anaerobic bacteria.

**Conclusions:**

This study represents the first comprehensive analysis of microaerophilic Chlamydomonadales algae. Having retained several ancestral enzymes of the anaerobic energy metabolism, *Leontynka* represents a unique vantage point for understanding the evolution of the hydrogen production machinery and adaptations to low oxygen in Chlamydomonadales (and core chlorophytes in general). Our findings suggest that the plastid of non-photosynthetic *Leontynka* follows a similar evolutionary path as mitochondria when adapting to anaerobiosis and parallels the transition of a mitochondrion into a hydrogenosome.

**Supplementary Information:**

The online version contains supplementary material available at 10.1186/s12915-026-02529-3.

## Background

The order Chlamydomonadales (Chlorophyceae, Chlorophyta) represents a diverse group of photosynthetic and non-photosynthetic microalgae. The former category is epitomized by *Chlamydomonas reinhardtii*, a model organism that can be manipulated using genetic and metabolic engineering methods [1] and has great potential for the green economy [2]. *C. reinhardtii* is highly metabolically flexible. It is capable of both photoautotrophic and heterotrophic metabolism [3] and grows in various oxygen concentrations, employing anaerobic energy production in low oxygen [4]. Metabolites produced by *C. reinhardtii* have various applications; e.g., it is a source of nutraceutical and food supplements [5] or biohydrogen [6]. The alga assimilates acetate [7] and degrades cellulose [8]. Some chlamydomonadaleans lost the ability to perform photosynthesis and rely exclusively on a heterotrophic metabolism [9–12]. In contrast to *C. reinhardtii*, the non-photosynthetic genus *Polytomella* can utilize alcohols and organic acids, such as butyrate, and does not survive in low oxygen concentrations [12]. However, *P. parva* is the only heterotrophic chlamydomonadalean whose energy metabolism has been studied in greater detail.

To better understand the variation and evolution of energy metabolism in heterotrophic Chlamydomonadales, we investigated the recently described non-photosynthetic genus *Leontynka*, which represents a deep-branching lineage, evolutionarily distinct from other non-photosynthetic members of the group. Two morphologically and genetically distinguishable species, *L. pallida* and *L. elongata*, were isolated from low-oxygen habitats [13]. Furthermore, the same study reported on the sequencing and analysis of the plastid and mitochondrial genomes of *L. pallida*, highlighting the extreme repeat content of both and providing interesting insights into a peculiar evolutionary trajectory followed by these organellar genomes. However, the metabolism and oxygen dependency of *Leontynka* spp. have not been investigated. In the current study, we complemented the previously reported transcriptome assembly from *L. pallida* by generating a high-quality transcriptome assembly from *L. elongata*. We explored these data to obtain an in silico reconstruction of the energy production pathways of each species, which we then compared with the metabolism of *C. reinhardtii* and *P. parva*. We supported our predictions with Raman microspectroscopy, high-performance liquid chromatography, growth experiments, and electron tomography to paint a complex physiological portrait of *Leontynka*.

## Results

### *Leontynka* thrives in microoxic conditions

**Fig. 1:**
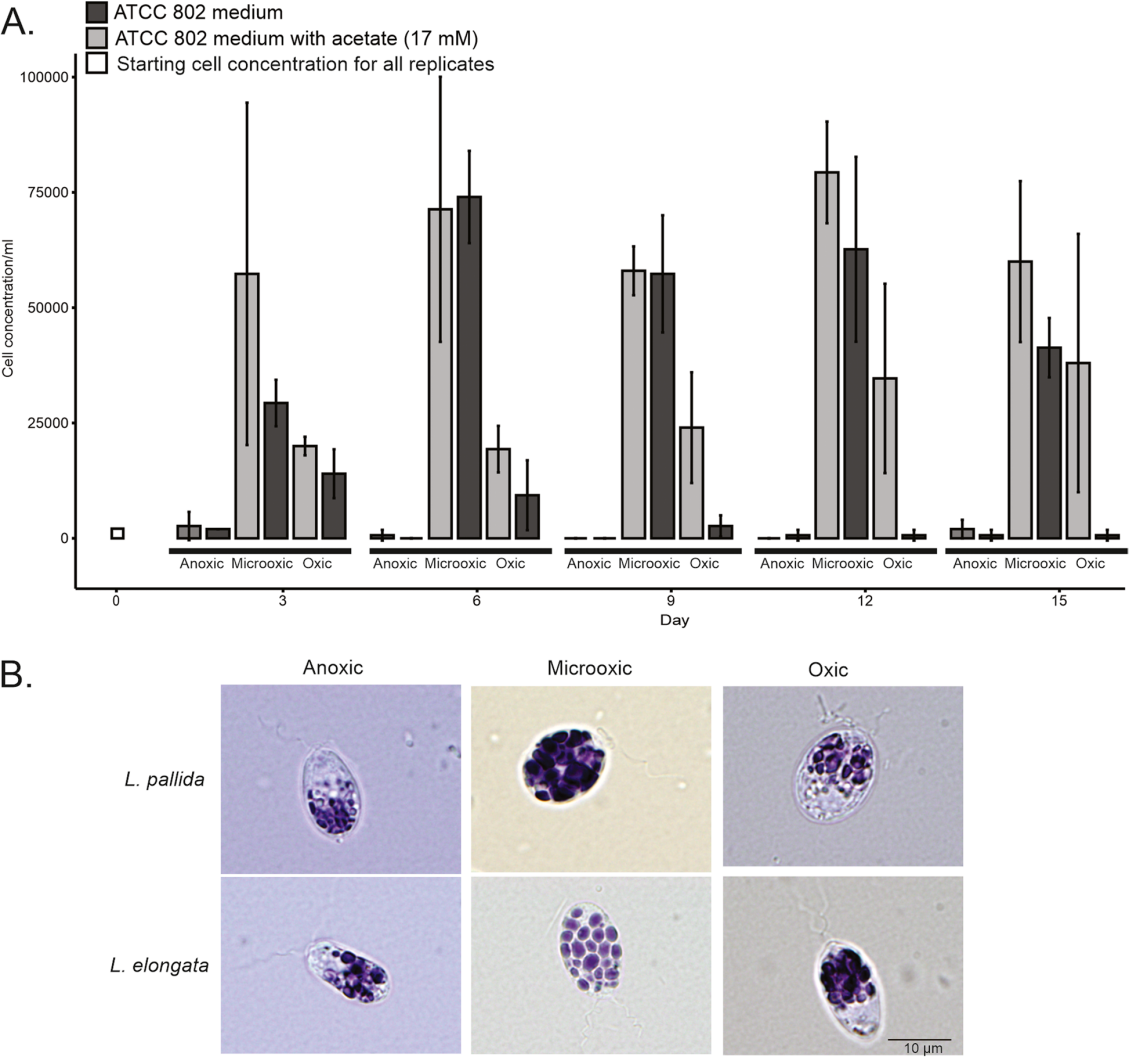
Growth of *Leontynka* spp. in different oxygen levels.** A** Cell densities of *L. pallida* polyxenic gnothobiotic culture grown in “anoxic” (< 1% O_2_), “microoxic” (8–9% O_2_), and “oxic” conditions (normoxia) in the presence or absence of added acetate. The starting amount was 1400 cells; three tubes (replicates) were established and analyzed for each condition. Only viable cells (identified by trypan blue staining) were counted. The error bars represent the standard deviation of the replicates for each condition per the time interval. **B** Abundance and morphology of starch granules in in *L. pallida* and *L. elongata* grown in three different oxygen levels for 8 days without added acetate. Starch granules were stained purple using Lugol’s solution and observed 8 days after inoculation

*Leontynka* spp. were originally isolated from freshwater hypoxic/microoxic sediments [13] namely a small freshwater body rich in organic sediments of plant origin. Such environments bring challenges, such as fluctuation of oxygen levels and lack of light to secure enough photons for photosynthesis. The oxygen level requirements or tolerance to anoxia have not been previously investigated in *Leontynka* spp. Hence, we performed growth experiments with *L. pallida* to test its ability to grow in various oxygen levels (see Additional file 1: Fig. S1). These experiments show that *L. pallida* thrives in microoxic conditions, while it survives in low densities in an anaerobic chamber and at normoxia (Fig. 1A). Cells of both *Leontynka* spp. appear to be smaller and contain fewer and smaller starch granules when grown in an anaerobic chamber (Fig. 1B). The addition of acetate stimulates the growth of *Leontynka* in normoxia, indicating that it utilizes acetate as a source of energy and/or carbon (Fig. 1A). However, cultures of both *Leontynka* spp. contain multiple prokaryotes, which were originally co-isolated from nature together with them. Consequently, their assembled (meta)transcriptomes contain sequences corresponding to prokaryotic RNAs (see Additional file 2: Tables S1 and S2). Based on this data, the composition of the prokaryotic community associated with both *Leontynka* spp. was thoroughly examined (see Additional file 1: Fig. S2). In short, both cultures each contain 13 prokaryotic lineages (16S rRNA gene sequences with the relative abundance < 1% were not considered as they likely represent contaminants, e.g., due to sample bleeding during sequencing). While the aerobic prokaryotic components of the two cultures do not overlap, the anaerobic and microaerophilic components are surprisingly similar, with the genus *Azospirillum* being a dominant lineage in both cultures at the moment of RNA extraction (7 days after inoculation). As we did not observe any endo- or ectosymbionts associated with the cells of *Leontynka* spp. using transmission electron microscopy (TEM; [13]), we assume that all these bacteria are free-living. As some of them utilize or produce acetate (see Additional file 1: Fig. S2), we cannot rule out the possibility that *Leontynka*’s growth after addition of acetate is stimulated by acetate-utilizing bacteria, rather than directly by acetate itself. Our attempts to establish axenic *Leontynka* cultures to distinguish between the two possibilities were unsuccessful.

### Building a resource for bioinformatic analyses of the *Leontynka* metabolic networks

To expand the sequence resources for investigating the biology of *Leontynka*, we complemented the previously published organellar genome sequences and transcriptome assembly from *L. pallida* [13] by generating a transcriptome assembly for *L. elongata* and then predicted the putative nucleus-encoded proteomes of both species. Their completeness was compared with reference data from other Chlamydomonadales (see Additional file 1: Fig. S3 and Additional file 3: Notes S1), which indicated that the data from *Leontynka* spp. are of high quality and provide a sufficiently comprehensive resource for exploring the metabolic capabilities of both species. Still, the analysis indicated that approximately 10% and 15% of proteins are missing from the predicted proteomes of *L. pallida *and* L. elongata*, respectively. With this limitation in mind, we exploited the data to in silico reconstruct a major part of the metabolic network of *Leontynka* spp., focusing on carbon and energetic metabolism. Notably, the newly generated data from *L. elongata* confirmed that this organism does not encode any component of the photosynthetic machinery and thus relies fully on heterotrophy. In addition to the enzymes previously reported as missing from *L. pallida* [13], we also revealed that *Leontynka* spp. lack key enzymes of the Calvin–Benson–Bassham cycle (CBB), namely ribulose-bisphosphate carboxylase and phosphoribulokinase, both of which are essential for photosynthetic carbon fixation.

The accuracy of any bioinformatic metabolic reconstruction also depends on the ability to predict the subcellular localization of the proteins analyzed. We took advantage of the extensive experimentally validated data on protein subcellular localization in *C. reinhardtii* (see Additional file 2: Table S3) [14–16] to benchmark available bioinformatic prediction tools in order to design a pipeline maximizing the prediction accuracy for *Leontynka* proteins (see Additional file 2: Tables S4–S7 and Additional file 3: Notes S2) [14–28]. As a result (assuming that the features defining specific organellar-targeting presequences are generally similar in *C. reinhardtii* and *Leontynka* spp.), we used a consensus of the three best-performing prediction tools as a conservative approach to minimize false positives. We thus expect a significant portion of plastid and mitochondrial proteins to stay unassigned. On the other hand, the probability that we assign a protein as mitochondrial when it is, in fact, localized to the plastid (or vice versa) is very low. To justify the presumption that *C. reinhardtii* and *Leontynka* spp. share features defining their organellar targeting presequences, we evaluated whether putative plastid and mitochondrial proteins from *Leontynka* spp. showed typical features of chloroplast transit peptides (cTP) or mitochondrial transit peptides (mTP) (see Additional file 3: Notes S2). Indeed, predicted *Leontynka* cTPs and mTPs show the expected charge distribution with cTPs being relatively uncharged at the N-terminus and most positively charged in the central part, and mTPs most positively charged at the very beginning (see Additional file 1: Fig. S4). Sentinel amino acids also follow the expected patterns (see Additional file 1: Fig. S5). We found a high prevalence of “GLK”-sites, putative interaction sites for the chloroplast protein import machinery, in the N-terminal ~ 40 residues of predicted plastid proteins, as well as in the first ~ 20 residues of predicted mitochondrial proteins, matching the situation in *C. reinhardtii* and providing an additional criterion to distinguish plastidial and mitochondrial proteins in *Leontynka* spp. [22, 27]*.*

### *Leontynka* is an osmotroph equipped with enzymes for cellulose degradation

**Fig. 2:**
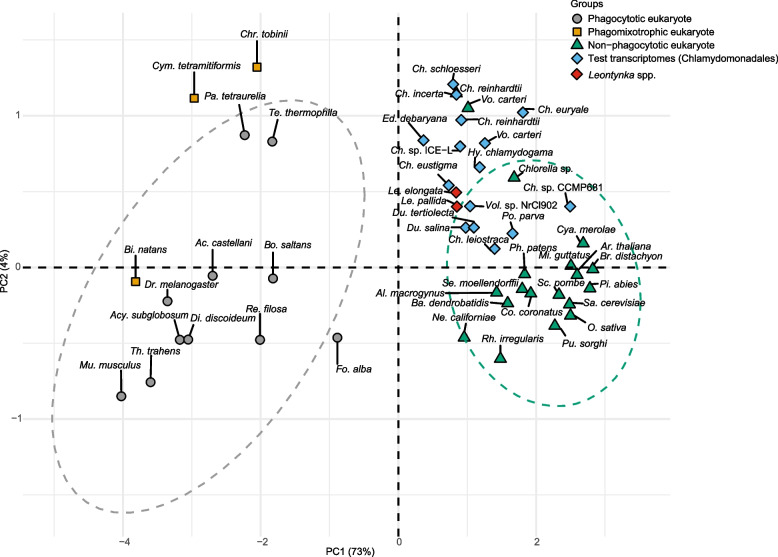
Trophic mode prediction of Chlamydomonadales including *Leontynka *spp. based on transcriptome predictive model using the tool predictTrophicMode. Most species depicted in the figure are available directly from the predictTrophicMode tool database. Database was enriched by predicted proteomes from Chlamydomonadales collected from EukProt v.3 deposited on Figshare [34]: *Hyalomonas chlamydogama* (EP00195), *Chlamydomonas euryale* (EP00955), *Ch. eustigma* (EP00799), *Ch. incerta* (EP00851), *Ch. leiostraca* (EP00197), *Ch. reinhardtii* (EP00198), *Ch. schloesseri* (EP00852), *Chlamydomonas* sp. CCMP681 (EP00199), *Chlamydomonas* sp. ICE − L (EP00798), *Dunaliella salina* (EP00874), *Du. tertiolecta* (EP00200), *Edaphochlamys debaryana* (EP00853), *Volvox carteri* (EP00202), and other databases: Chlamydomonadales sp. NrCl902 [35] deposited in the NCBI SRA archive (reads assembled and proteins predicted de novo), *Polytomella parva* downloaded from iMicrobe [36], and *Leontynka pallida* from Figshare [37]. The predicted proteome of *Leontynka elongata* was generated in this study

Heterotrophic metabolism of green algae—both facultative and obligate—is generally based on osmotrophy, i.e., the uptake of dissolved organic compounds from the extracellular environment via membrane transporters [29, 30], whereas phagotrophy has been documented only from several prasinophytes as part of a mixotrophic lifestyle [31, 32]. Indeed, none of the *Leontynka* and other Chlamydomonadales species analyzed with the predictTrophicMode tool [33] were classified as phagotrophs (Fig. 2). Specifically, both *Leontynka* spp*.* showed low phagocytotic probability scores (0.01), clustering closely with non-phagocytotic eukaryotes. This indicates that *Leontynka* spp., like other non-photosynthetic Chlamydomonadales, are osmotrophs, an unsurprising conclusion also given the presence of a thick cell wall (“chlamys”) that encloses almost the whole surface of the cell.

**Fig. 3:**
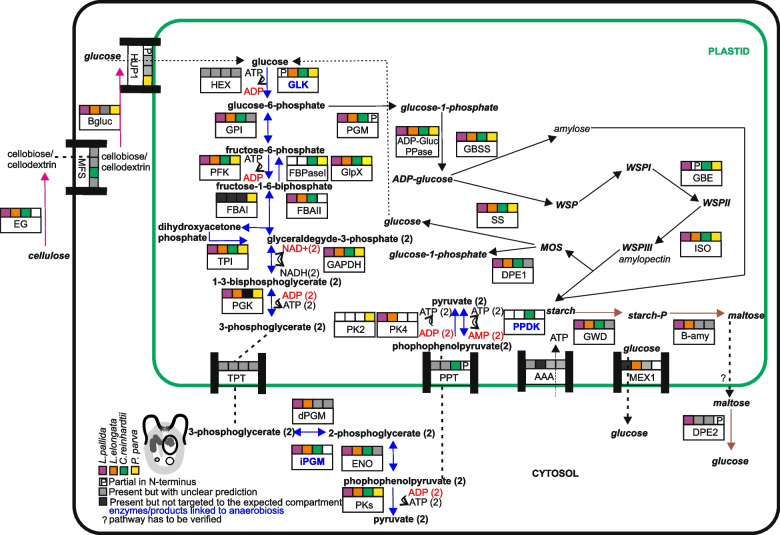
Schematic map of carbohydrate metabolism and starch formation in *Leontynka *spp. and other Chlamydomonadales. The scheme indicates the presence/absence and (putative) subcellular localization of relevant enzymes for four species, *Leontynka pallida*, *L. elongata*, *Chlamydomonas reinhardtii*, and *Polytomella parva* (see the graphical legend in the bottom left corner). Enzymes are assigned to specific subcellular compartments based on all available data, including publicly available experimental results. Squares filled with color indicate that our in silico localization predictions are consistent with the depicted localization, either as expected or experimentally verified, in *C. reinhardtii*. Membrane and peroxisomal proteins are determined only by DeepLoc v2.1, so if a given protein was categorized as “other” in other two programs, the DeepLoc prediction was considered correct. The same legend applies to all metabolic schemes in this study. Written in italics are compounds involved in starch synthesis and degradation. Abbreviations: Endo/exoglucanase (EG); Major facilitator superfamily (MFS); ß−1,4-glucosidase (Bgluc), H(+)/hexose cotransporter 1 (HUP1); hexokinase (HEX); glucokinase (GLK); glucose-6-phosphate isomerase (GPI); phosphofructokinase (PFK); fructose-1,6-bisphosphate aldolase class I (FBAI); fructose-1,6-bisphosphate aldolase class II (FBAII); triosephosphate isomerase (TPI); glyceraldehyde-3-phosphate dehydrogenase (GAPDH); phosphoglycerate kinase (PGK); triosephosphate/inorganic phosphate translocators (TPP); cofactor-dependent phosphoglycerate mutase (dPGM); cofactor-independent phosphoglycerate mutase (iPGM); enolase (ENO), pyruvate kinase (PK); pyruvate, phosphate dikinase (PPDK); phosphoenolpyruvate translocators (PPT); ATP/ADP transporter (AAA); fructose-1,6-bisphosphatase class I (FBPaseI); fructose-1,6-bisphosphatase class II, GlpX-type (GlpX); phosphoglucomutase (PGM); ADP-glucose pyrophosphorylase (ADP-Glc PPase); granule-bound starch synthase (GBSS); starch synthase (SS); 1,4-alpha-glucan branching enzyme (GBE); isoamylase (ISO); disproportionating enzyme 1 (DPE1); glucan water dikinase (GWD); beta-amylase (B-amy); maltose exporter-like protein 1 (MEX1); disproportionating enzyme 2 (DPE2). Written in red are oxidized molecules such as NAD^+^ and dephosphorylated molecules such as ADP and AMP. The number two enclosed in parentheses represents the number of molecules involved in the reaction. Source data deposited in Additional file 2: Tables S8–S11

The predicted osmotrophy of *Leontynka* spp. is also in accordance with our detailed reconstruction of their metabolic capabilities. Firstly, *Leontynka* spp. utilize extracellular cellulose, degrading it to glucose in a pathway similar to that described in *C. reinhardtii* (Fig. 3, see Additional file 3: Notes S3) [8]. Cellulases of *L. pallida* and *L. elongata* are predicted as extracellular and constitute a deep-branching clade among Chlamydomonadales homologs, together with them nested among homologs from other Chlorophyta (see Additional file 1: Fig. S6). We also found a cytoplasmic β-glucosidase, an enzyme responsible for the final step of the whole pathway that converts cellobiose to glucose [38]. Its substrate specificity to cellobiose is supported by characteristic amino acid residues previously defined in the *Thermoanaerobacter brockii* enzyme (see Additional file 1: Fig. S7). Using Raman microscopy, we did not detect cellulose in the cell wall in any of the 40 measured cells in the early (36 h) or the late exponential state (7 days). This is consistent with data from the cell wall of the *C. reinhardtii*, which is composed of glycoprotein-rich layers [39]. Furthermore, no cellulose signal was detected inside the *Leontynka* cell. Hence, *Leontynka* does not have any internal cellulose-based structures that would be degraded by its cellulases, further supporting the deployment of *Leontynka* cellulases for catabolizing external cellulose sources.

### *Leontynka* accumulates diverse storage compounds

**Fig. 4:**
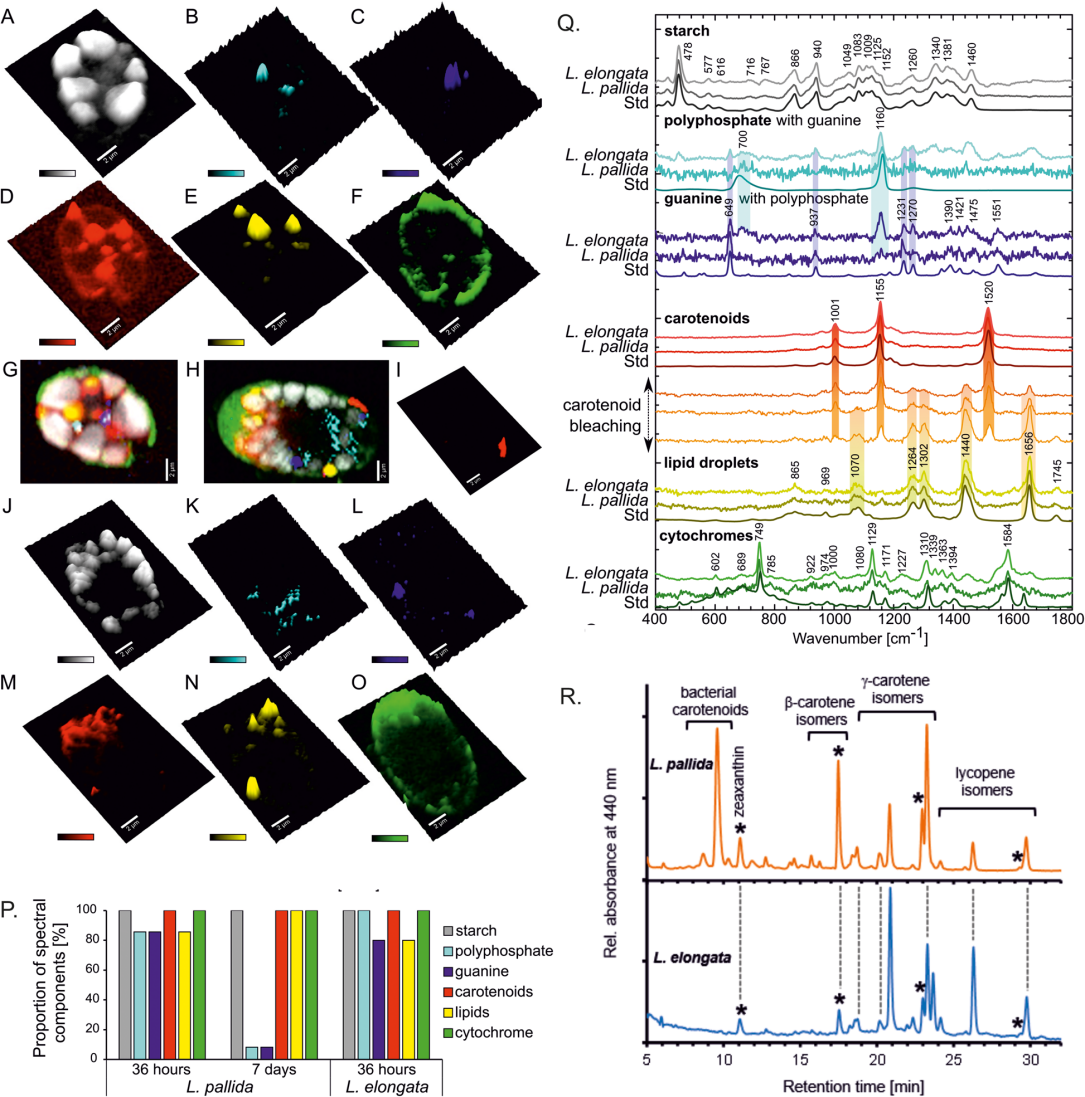
Raman spectral imaging of *L. pallida* (A–G) and *L. elongata* (H–O) measured after 36 h of growth. Chemical maps depict normalized intensities of major spectral components in pseudo-3D representation: **A**, **J** Starch granules of α−1,4-glucan. **B**, **K** Polyphosphate. **C**, **L** Guanine. **D**, **I**, **M** Carotenoids. **E**, **N** Lipid droplets formed by triacylglycerols. **F**, **O** Cytochromes. **G**, **H** Merged 2D map. I Eyespot. Scalebar: 2 µm. **P** Distribution of the major spectral components highlighting the storage compartments (*n* = 24, 5–12 per time point). Videos are shown in Additional file 4. **Q** Raman spectra of the major spectral components in the two species and the chemically pure standard, with a special emphasis on the colocalized guanine and polyphosphate and the process of photobleaching of carotenoids present in lipid droplets containing triacylglycerols. **R** HPLC chromatograms of carotenoid extracts of *Leontynka* spp. Asterisks denote the peaks of all-trans isomers of carotenoids. Pigment extracts from bacterial cells and the culture medium are shown in Additional file 1: Fig. S10

We employed various staining techniques and polarization microscopy to visualize components in *Leontynka* cells (see Additional file 1: Fig. S8) and Raman microspectroscopy for label-free chemical imaging of the major storage compartments in both *L. pallida* and *L. elongata* (Fig. 4, see Additional file 4). Both species showed a comparable cell composition with some variability depending on the physiological state of the culture during its growth. We identified four types of storage species, namely starch granules (Fig. 4A, J, see Additional file 1: Fig. S8A,C,E), polyphosphate (Fig. 4B, K, see Additional file 1: Fig. S8D), guanine crystals (Fig. 4C, L, see Additional file 1: Fig. S8F), and lipid droplets (Fig. 4E, N, see Additional file 1: Fig. S8H). While guanine crystals serve as a storage of organic nitrogen [40], polyphosphate serves as a storage of both phosphorus and energy [41] and starch and lipids provide both energy and organic carbon for anabolic reactions [42]. Starch accumulates in large granules inside the plastids of *Leontynka* (Figs. 1B and 4A, J), as suggested previously based on light microscopy and TEM [13]. Most likely, it serves as an energy reserve under nutrient-deficient conditions, as is typical for other algae [43]. We recovered homologs of all enzymes involved in the synthesis and degradation of starch in *Leontynka* spp. (Fig. 3).

Arguably, lipid droplets and starch might persist as products of the stationary growth phase before reinoculation. During the lag phase or early exponential phase (36 h after inoculation), cells contained all storage compartments at once in both *Leontynka* species (Fig. 4P). Polyphosphate granules and guanine crystals occasionally co-localized in a single compartment, as documented on the chemical maps as well as the Raman spectra depicting both components in a single voxel (Fig. 4B,C,K,L,Q). Such co-localization has been previously spotted in the versatile vacuoles of other green algae [44, 45]. Both usually appear during the lag phase as a result of a “luxury uptake” after the nutrients are resupplied following the period of starvation. Later, they are consumed to support biosynthesis of nucleic acids and proteins [40, 44, 46]. In line with the Raman signal for polyphosphate, we found in *Leontynka* spp. homologs of the vacuolar transporter chaperone complex subunit 4 (VTC4) gene required for polyphosphate synthesis and of genes for regulatory proteins that influence the accumulation of polyphosphate (see Additional file 2: Tables S8 and S9) [47, 48].

Despite the loss of photosynthesis, the lipid metabolism in *Leontynka* spp. fits the situation in *C. reinhardtii* (see Additional file 1: Fig. S9). Specifically, we identified all enzymes required for the plastid-localized biosynthesis of fatty acids as well as their catabolism in peroxisomes via the β-oxidation pathway. *Leontynka* spp. have retained the enzymes for the biosynthesis of plastids-specific structural lipids, namely galactolipids and sulfoquinovosyldiacylglycerol, which is notable, as these pathways (one or both) are frequently missing from non-photosynthetic plastids of other eukaryotes [49, 50]. We also identified both enzymes that are responsible for the production of triacylglycerols (TAGs) stored in lipid droplets in *C. reinhardtii*, namely diacylglycerol acyltransferase and galactolipid lipase [51, 52]. The spectral fingerprint of TAGs of *Leontynka* spp. indicated the content of unsaturated fatty acids. Counting the ratio of intensities of C = C double bond vibrations and CH_2_ vibrations (the respective wavenumbers being 1656 and 1440 cm^−1^) yields the ratio of 1.07 ± 0.29 (*n* = 20 particles), reflecting monounsaturated fatty acids contents according to Czamara et al. [53]. The overall proportion of double bonds in *Leontynka* TAGs is up to 1.8, corresponding to an increased level of polyunsaturated fatty acids, e.g., linoleic (18:2) and α-linolenic (18:3) compared to monounsaturated, e.g., palmitoleic (16:1) and oleic (18:1), and saturated (e.g., palmitic (16:0) and stearic (18:0)) fatty acids. This pattern is comparable to other green algae but more prominent in *Leontynka* spp. compared to *C. reinhardtii* [54, 55].

### *Leontynka* synthesizes only carotenoids with β-rings and preferentially accumulates *cis*-carotenoids

Isoprenoids are a diverse group of compounds with many key roles in the cell, synthesized from the common precursor isopentenyl pyrophosphate (IPP). In *C. reinhardtii* and chlorophytes in general, IPP is produced exclusively by the plastidial methyl-D-erythritol 4-phosphate (MEP) pathway [56, 57], and unsurprisingly, we reconstructed the complete MEP pathway in *Leontynka* spp., too (see Additional file 2: Tables S8 and S9). One of the major isoprenoid categories comprises carotenoids. These were revealed by Raman measurements to be present in a high proportion in the lipid droplets in *Leontynka*. In fact, they were so abundant as to fully obscure the Raman signal of TAGs, which was revealed only after thorough photobleaching of carotenoids (Fig. 4Q). Carotenoid Raman signal enhancement by several orders of magnitude is based on their effective Raman cross-section [58], yet simultaneously, carotenoids are susceptible to photodamage or photobleaching. Thus, we optimized the measurement process over multiple steps (see Methods). We detected carotenoids also in the plastid membranes encapsulating the starch granules (Fig. 4A, D, J, M; in the latter case we induced unintentional photobleaching of the lower part of the cell losing a subtle carotenoid signal from both the plastid membranes and lipid droplets). The presence of carotenoids in *Leontynka* plastid membranes suggests their structural or functional role in maintaining membrane fluidity and stability as well as providing antioxidant capacity [59]. Carotenoids are also the major component of the eyespot localized in the *Leontynka* plastid (Fig. 4I).

Since Raman microspectroscopy is not sensitive enough to determine the identity of the observed carotenoids, we performed high-performance liquid chromatography (HPLC) analysis, which detected the *trans*- and various *cis-*isomers of β-carotene, γ-carotene, lycopene, and small amounts of *trans*-zeaxanthin in both *Leontynka* species (Fig. 4R). The pigment extracts from *L. pallida* cultures also contained three unknown polar carotenoids synthesized by the associated bacterial microbiota as shown by HPLC analysis of a sample strongly enriched in the bacterial fraction (see Additional file 1: Fig. S10). The major peak eluting at 9.7 min may represent nostoxanthin, as its known source *Novosphingobium* [60] is also present in the *L. pallida* culture. In both *Leontynka* cultures, we found a phototrophic bacterium affiliated to the genus *Rhodocyclus*, but we did not detect bacteriochlorophyll *a* or carotenoids typical for these bacteria in the pigment extracts from the cultures. *Cis*-isomers of β-carotene, ϒ-carotene, and lycopene dominated in *Leontynka* spp., while *trans*-isomers were diminished and carotenoids with ε-ring were missing. We also detected minor amounts of zeaxanthin in both species, in agreement with our in silico analyses that also identified transcripts for β-carotene hydroxylase (CHYB), which converts β-carotene to zeaxanthin (see Additional file 1: Fig. S11). The preferential accumulation of putative *cis*-carotenes resembles observations reported for the carotenoid composition in eyespots of *C. reinhardtii* [61] and may be partially explained by the absence of carotenoid isomerase (CRTISO), which prevents the efficient formation of *trans*-lycopene and carotenoids derived thereof.

*Leontynka* spp. also lack lycopene ε-cyclase responsible for adding ε-rings to lycopene for lutein formation [62]. The loss of the ability to produce this xanthophyll is rationalized by lutein being primarily linked to photosynthesis as an integral component of light-harvesting complexes [63]. We also did not find any of the enzymes involved in producing violaxanthin and neoxanthin. We identified, however, a putative transcript of β-carotene ketolase (BKT) that is responsible for the production of secondary ketocarotenoids in other green algae, although we did not detect any ketocarotenoids in *Leontynka* spp. by HPLC. Detailed analyses revealed that the BKT transcripts from *Leontynka* spp. do not encode functional enzymes and likely represent remnants of the original BKT genes that evolved into pseudogenes (see Additional file 1: Fig. S12) [64].

### *Leontynka* requires oxygen for sterol synthesis

Besides carotenoids, another major isoprenoid category corresponds to sterols, key components of cell membranes in eukaryotes that are synthesized from squalene through a complex pathway that requires molecular oxygen. In *C. reinhardtii*, sterol synthesis occurs in the endoplasmic reticulum and starts with epoxidation of squalene performed by the flavoprotein squalene epoxidase (one of the O_2_-dependent steps of sterol biosynthesis), followed by cyclization to cycloartenol by the oxygen-independent enzyme cycloarthenol cyclase [65]. Both enzymes, as well as the enzymes catalyzing the following oxygen-dependent steps, namely obtusifoliol 14α-demethylase (CYP51), Sterol-C₄-methyl oxidase (SMO), Δ^7^-sterol-C_5_-desaturase (STE1), and sterol C-22 desaturase (CYP710) [66], are all present in *Leontynka* spp. (see Additional file 2: Tables S8 and S9). Instead of sterols, some eukaryotes produce from squalene different types of membrane-modulating triterpenoids, whose synthesis does not require oxygen. These include tetrahymanol synthesized by squalene-tetrahymanol cyclase (STC) [67] and hopanoids depending on squalene–hopene cyclase (SHC) [68]. We found neither STC nor SHC in *Leontynka*, implying that it requires at least a minimal level of O_2_ to ensure the proper function of its membranes.

### Glycolysis generates ATP in the *Leontynka* plastid

In contrast to most other eukaryotes, the first six steps of glycolysis are performed in the plastid of Chlamydomonadales. This configuration was experimentally demonstrated in *C. reinhardtii* [69] and supported to be valid for the whole group, including both *Leontynka* spp., by in silico predictions of the subcellular localization of the respective enzymes (Fig. 3). An important question here is which glyceraldehyde-3-phosphate dehydrogenase (GAPDH) isoforms actually participates in glycolysis in *Leontynka*. GAPDH is encoded by three genes (GAP1–GAP3) in *Chlamydomonas reinhardtii*. Both GAP1 and GAP3 contain N-terminal presequences that are clearly predicted to correspond to plastid-targeting signals (see Additional file 2: Table S10). This is consistent with their experimentally verified localization in the plastid stroma [16]. GAP3 is considered NAD(P)H-dependent and participates in the CBB cycle [70], whereas the cofactor specificity and functional role of GAP1 remain less certain. GAP3 and GAP2 are represented only by incomplete or unspliced transcript fragments in *Leontynka* spp. transcriptome assemblies, while GAP1 shows a much stronger transcriptomic representation and appears to be the dominant GAPDH version in both *Leontynka* species, possessing a clear plastid-targeting presequence (see Additional file 2: Tables S8 and S9). The low abundance of the possibly non-functional GAP3 in the transcriptomic data from *Leontynka* spp. is consistent with loss of the CBB. Our in silico analysis performed using the Rossmann-toolbox server [71] indicated that this enzyme is NAD⁺-dependent at least in *L. pallida* (confidence = 0.99). Therefore, we infer that GAP1 functions as the glycolytic GAPDH within the plastid of *Leontynka* spp.

The fate of NADH produced by the GAPDH reaction then differs depending on the availability of O_2_. In normoxia, it is apparently reoxidized by a plastid-targeted type II NADH dehydrogenase found in both *Leontynka* spp. (see Additional file 2: Tables S8 and S9), following the paradigm previously established for *C. reinhardtii* [72]. The electrons are passed to plastoquinone (reducing it to plastoquinol) and eventually to O_2_ by the plastid terminal oxidase (PTOX). The presence of plastoquinone and PTOX was previously noticed in *L. pallida* and their retention in the non-photosynthetic plastid of this protist was rationalized by pointing to the fact that *L. pallida* has preserved the carotenoid biosynthesis pathway (to supply carotenoids for its eyespot, see above), which includes two desaturase reactions feeding electrons into the plastid electron transport chain [13]. Our extended analysis indicated that this functional module is in fact linked to glycolysis in *L. pallida*, and identification of homologs of all the relevant enzymes in *L. elongata* (see Additional file 2: Tables S8 and S9) documents the conservation of this metabolic wiring in the genus *Leontynka* as a whole. Reoxidation of NADH generated by glycolysis in oxygen shortage is dissected in the subsequent section.

3-phosphoglycerate produced by the plastid-localized part of glycolysis is exported to the cytosol by a triose phosphate/inorganic phosphate transporter, where the remaining glycolysis steps are performed to produce pyruvate. In *C. reinhardtii*, the phosphate/phosphoenolpyruvate translocator transports a fraction of phosphoenolpyruvate (PEP) back into the plastid, where plastid-targeted pyruvate, phosphate dikinase (PPDK) catalyzes the reversible conversion of PEP to pyruvate [15, 72]. We did not find a PPDK transcript in the transcriptomic data from *Leontynka* spp., but both *Leontynka* spp. contain (and express) several paralogs of its functional alternative, pyruvate kinase (PK), some of which are predicted to be plastidial (see Additional file 2: Tables S8 and S9), in contrast to *C. reinhardtii* (see Additional file 2: Table S10). PK catalyzes an irreversible reaction, generating ATP and pyruvate. Altogether, this suggests that *Leontynka* can generate ATP and pyruvate within the organelle from imported PEP.

PPDK is used in *C. reinhardtii* and other eukaryotes to synthesize PEP from pyruvate as part of gluconeogenesis, so whether and how this step is performed in *Leontynka* spp. is uncertain, as the alternative enzyme (pyruvate, water dikinase) could also not be identified in their transcriptome data. We note that this reaction is not needed for assimilation of acetate, as *Leontynka* spp. can form PEP also from C4 acids generated via the glyoxylate cycle from C2 compounds thanks to possessing a complete glyoxylate cycle and a plastidial phosphoenolpyruvate carboxykinase (PEPCK; see Additional file 2: Tables S8 and S9). The gluconeogenetic conversion of fructose-1,6-bisphosphate to fructose 6-phosphate in *C. reinhardtii* proceeds by the action of class I fructose bisphosphatase (FBPaseI). Surprisingly, we did not identify FBPaseI in *Leontynka*. Instead, our search for alternative enzymes resulted in the discovery of a class II FBPase, also known as GlpX, which catalyzes the same reaction in bacteria [73]. Our comprehensive phylogenetic analysis showed that GlpX is present in a wide range of chlorophytes (including *C. reinhardtii* and prasinophytes) and was likely acquired by horizontal gene transfer from Actinomycetes before the radiation of the group (see Additional file 1: Fig. S13). The presence of a plastid-targeting signal in the *Leontynka* spp. GlpX and identification of the enzyme in the plastid proteomes of *C. reinhardtii* and *P. parva* [15, 74] provided additional support to our conclusion that GlpX has replaced the ancestral FBAseI in *Leontynka*.

### Krebs cycle and OXPHOS are fully operational in *Leontynka*

**Fig. 5:**
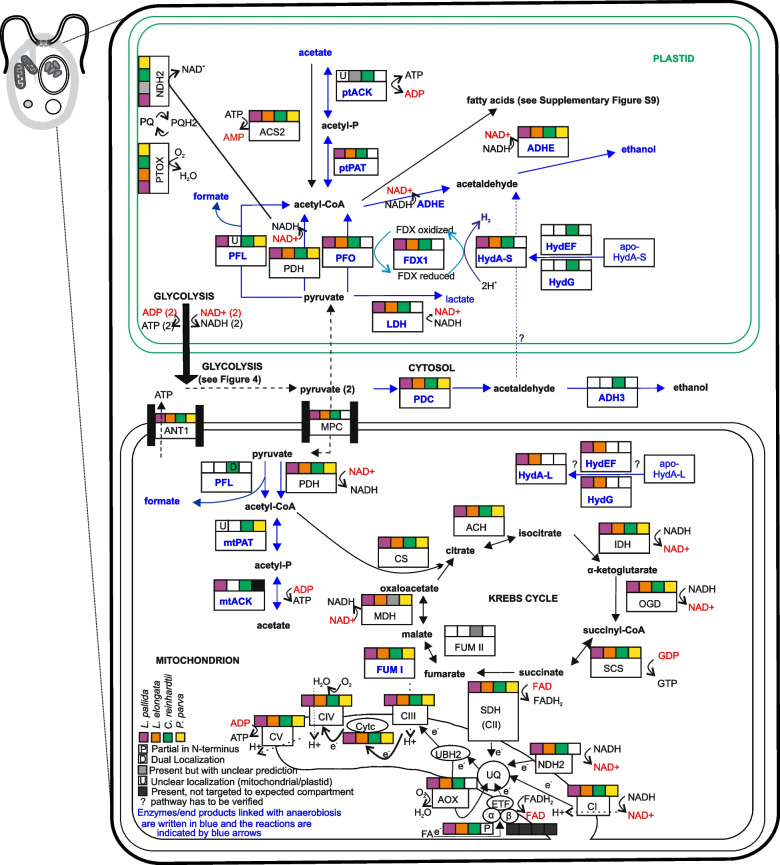
Reconstruction of the organellar anaerobic energy metabolism, Krebs cycle, and oxidative phosphorylation of *Leontynka *and other Chlamydomonadales. The scheme displays the presence/absence and (putative) subcellular localization of relevant enzymes for the same four species as Fig. 4, following the same display conventions (see also the graphical legend in the bottom left corner). Abbreviations: plastoquinone (PQ); plastoquinol (PQH2); plastid terminal oxidase (PTOX); type-II NADH dehydrogenase (NDH2); pyruvate dehydrogenase (PDH); pyruvate formate-lyase (PFL); acetyl-CoA synthetase (ACS2); phosphate acetyltransferase (PAT); pyruvate:ferredoxin oxidoreductase (PFO); FDX1 member of the ferredoxin family (FDX1); FeFe hydrogenase A (HydA; S-short; L-long); hydrogenase assembly factor G (HydG); hydrogenase maturase (HydEF); aldehyde/alcohol dehydrogenase ADH1/ADHE (ADHE); lactate dehydrogenase (LDH); pyruvate decarboxylase (PDC); acetaldehyde dehydrogenase (ADH3); mitochondrial pyruvate carrier (MPC); citrate synthase (CS); aconitate hydratase (ACH); isocitrate dehydrogenase (IDH); oxoglutarate dehydrogenase (ODG); succinyl-CoA ligase (SCS); succinate dehydrogenase (SDH); fumarate hydratase; class I (FUM I); fumarate hydratase; class II (FUM II); malate dehydrogenase NAD (MDH3); complex I (CI); alternative oxidase (AOX); electron-transfer flavoprotein dehydrogenase (ETF); electron transfer flavoproteins (α/β); ubiquinone (UQ); ubiquinol (UBH2); cytochrome *c* (Cytc); complex II (CII); complex III (CIII); complex IV (CVI); complex V (CV). Written in red are oxidized molecules such as NAD^+^ and FAD and dephosphorylated molecules such as ADP, GDP and AMP. The number two enclosed in parentheses represents the number of molecules involved in the reaction. Source data deposited in Additional file 2: Tables S8–S11

**Fig. 6:**
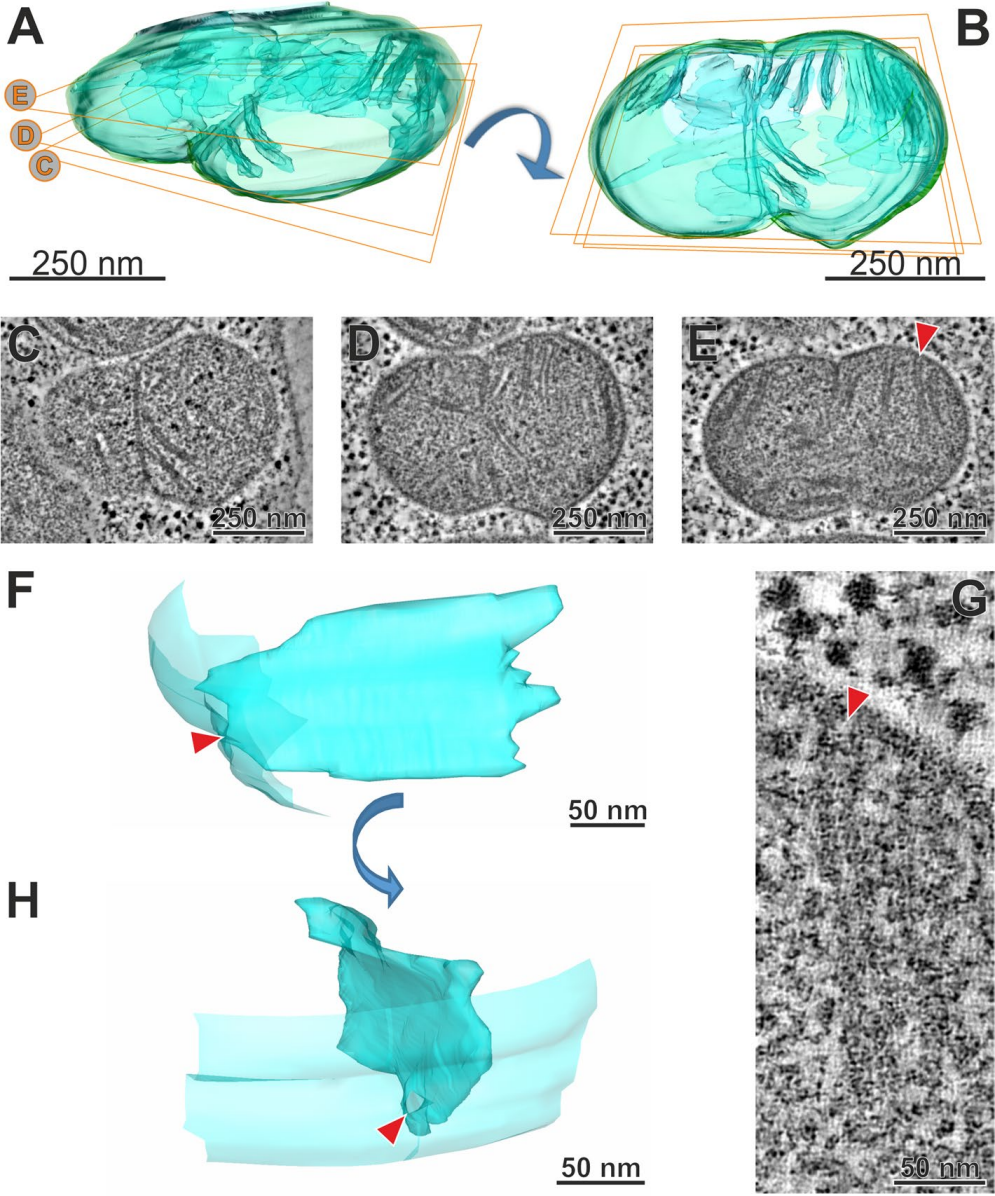
Discoidal cristae in *L. elongata*. Panels** A** and **B** show a model of a dividing *Leontynka* mitochondrion created by segmentation from an electron tomography reconstruction with an isotropic pixel size of 0.67 nm. Panels **C**, **D**, and **E** are sections with a thickness of 0.67 nm from different depths of the model/tomogram depicted in panels **A** and **B**. The red arrow marks the crista junction of the representative crista (model of the whole crista in panels **F** and **H**, tomogram detail shown in panel **G**)

*Leontynka* exhibits the full set of enzymes for the conventional pyruvate metabolism under normoxia (see above). Both *Leontynka* species have a fully operational Krebs cycle, electron transport chain, and mitochondrial ATP synthase, and are thus capable of oxidative phosphorylation (OXPHOS) (Fig. 5). Because OXPHOS requires mitochondrial cristae [75], we employed serial electron tomography to thoroughly investigate cristae morphology in *Leontynka*. We reconstructed 16 complete cristae in a dividing *L. elongata* mitochondrion (Fig. 6). They are indeed well-developed and all fitting the definition of the discoidal morphotype [76]. Disc-like cristae sometimes possess a pedicel, but this is not the case with *L. elongata* (Fig. 6F). The measured dimensions in the widest and highest part of the cristae were 182.2 ± 71.5 nm and 116.0 ± 25.7 nm (*n* = 16), respectively. Each crista communicates with the intermembrane space via a single crista junction (CJ; Fig. 6F–H), which is circular, 21 nm in diameter (*n* = 25; see Additional file 1: Fig. S14). Details on the *L. elongata* crista morphology, CJs, and comparison with other available data from discoidal cristae are summarized in Additional file 3: Notes S4 [76, 77]. Using ultrastructural data, we also investigated the position of mitochondria inside 11 cells of *L. elongata* and revealed their concentration beneath the cell membrane (see Additional file 1: Fig. S15 and Additional file 2: Tables S12 and S13). The localization of mitochondria corresponded with the Raman signal for cytochromes (Fig. 4F, O). This signal was one of the major spectral components of *L. elongata* cell, in contrast to the reports on *C. reinhardtii* using similar methods where the cytochrome signal is lacking [78]. This indicates that OXPHOS is an important route of energy metabolism in *Leontynka*.

### Enzymes underpinning the anaerobic energy metabolism in *Leontynka* have deep evolutionary roots

In agreement with the observed ability of *Leontynka* spp. to thrive under a limited O_2_ supply, they are equipped with enzymes mediating anaerobic energetic metabolism, with pyruvate as the starting point (Fig. 5). One route is the conversion of pyruvate into lactate by plastidial lactate dehydrogenase to regenerate NAD^+^ consumed during glycolysis. The other route regenerates NAD^+^ via ethanol fermentation. In *C. reinhardtii* the major ethanol-producing enzyme at dark anoxia is the plastidial bifunctional aldehyde/alcohol dehydrogenase (ADHE) [79]. It operates on acetyl-CoA (produced from pyruvate) or on acetaldehyde. In *C. reinhardtii*, acetaldehyde produced by pyruvate decarboxylase (PDC) in the cytosol can also be converted to ethanol by cytosolic alcohol dehydrogenase ADH3. While the latter enzyme is missing in *Leontynka* spp., ADHE is present in a single copy in both species and is predicted as plastidial (see Additional file 2: Tables S8 and S9). The normal functionality of the *Leontynka* ADHE is supported by the presence of conserved signature motifs involved in iron binding and of a tyrosine residue essential for the enzyme activity [80]. In contrast, the *P. parva* ADHE lacks the tyrosine residue [81].

*C. reinhardtii* encodes two types of organelle-targeted enzymes that metabolize pyruvate to acetyl-CoA in anaerobic conditions. One is pyruvate formate lyase (PFL), encoded by a single-copy gene and predominantly present in the mitochondrion, with a weaker signal also in the plastid. It converts pyruvate to acetyl-CoA and formate and is very active under dark anoxia [82]. The other one, pyruvate:ferredoxin oxidoreductase (PFO), is localized in the *C. reinhardtii* plastid where it is linked to the generation of H_2_ as a waste product [83]. To illuminate the evolutionary history of these key enzymes of anaerobic ATP generation in a broader phylogenetic context, we conducted a comprehensive search and phylogenetic analysis of these enzymes across Chlamydomonadales and their relatives. Our phylogenetic analyses showed that PFL and PFO are widely distributed across core chlorophytes, were most likely acquired prior to their radiation (PFL) or at least no later than after the divergence of Pedinophyceae from other core chlorophytes (PFO), and have a single origin (see Additional file 1: Figs. S16 and S17). Besides, PFL was subject to multiple lineage-specific duplications in Chlamydomonadales, with some of the copies likely retargeted to different cell compartments (see Additional file 1: Fig. S18).

Notably, among the non-photosynthetic Chlamydomonadales, only *Leontynka* spp. have retained both ancestrally present PFL and PFO enzymes. *L. pallida* contains a single pair of PFL and its activating enzyme (PFLA), with both proteins predicted as plastidial. Moreover, their N-termini match the sequence pattern expected for plastid-targeted proteins. On the other hand, the localization of the two and three distinct PFL and PFLA copies, respectively, encoded by *L. elongata* is unclear, as their N-termini follow neither a mitochondrial nor a plastidial pattern (see Additional file 2: Table S16).

**Fig. 7:**
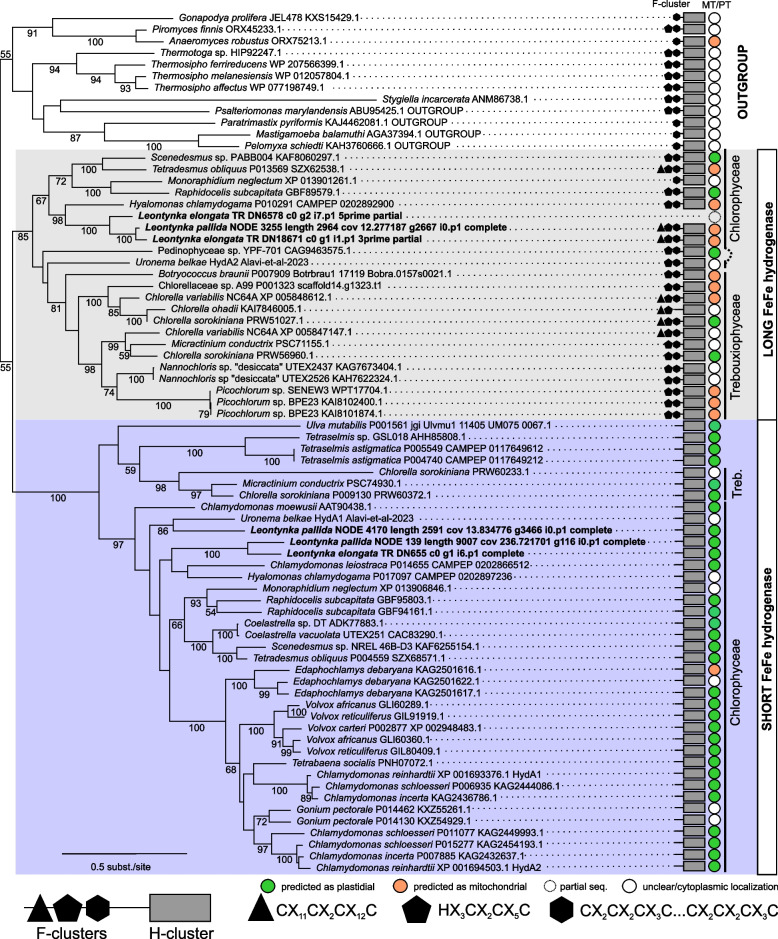
Phylogeny of FeFe hydrogenases in core chlorophytes, predicted localization, and distinctive features. The tree was computed using IQ-TREE (LG4X model; 300 bootstrap replicates) and visualized using ITOL. The dataset included 74 protein sequences and 426 amino acid positions after trimming. It contains all complete FeFe hydrogenases identified in core chlorophytes in this study, along with 12 sequences from other eukaryotes as an outgroup. The presence of motifs typical for the F-cluster (specified in the graphical legend) is indicated on the right and mapped onto the phylogeny of FeFe hydrogenase. The colors of the circles represent predicted subcellular localization. Sequences obtained from *Leontynka* spp. are in bold (N-terminus of one *L. pallida* homolog is incomplete, the sequence was included for exhaustiveness). Statistical support values below 50 are not shown. Source data are deposited in Additional file 2: Table S14

Acetyl-CoA production by PFO (but not PFL) is linked to the reduction of low-potential ferredoxins. In fact, the *C. reinhardtii* plastid harbors several ferredoxins, but only ferredoxin 1 (FDX1, also called PETF; see Additional file 1: Fig. S19) works with a short FeFe hydrogenase (HydA-S) [83], being reoxidized while H_2_ is emitted. Similarly, the plastid of *Leontynka* is predicted to harbor the complete PFO/FDX1/HydA-S machinery (Fig. 5). The HydA-S in *Leontynka* possesses the canonical amino acid residues R171 and K396 that are important for interaction with FDX1 [84–86]. Therefore, we conclude that *Leontynka* spp. can metabolize pyruvate to acetyl-CoA in the plastid, concomitantly producing H_2_. While *L. elongata* possesses one and *L. pallida* two homologs of plastidial HydA-S, *C. reinhardtii* possesses two (HydA1 and HydA2), and *Polytomella parva* contains none. Interestingly, besides HydA-S containing only the catalytic center (H-cluster) and predicted as plastidial, *Leontynka* also possesses a canonical (long) FeFe hydrogenase that has retained F-clusters at the N-terminus (HydA-L) and is predicted as mitochondrial. This makes *Leontynka* a rare case among Chlamydomonadales, although our phylogenetic analyses clearly show that the coexistence of HydA-S and HydA-L is the ancestral state for Chlamydomonadales and Chlorophyceae as a whole, as indicated by the taxon composition of both well-supported clades and by the coexistence of the two forms in a few taxa (see Fig. 7). The predicted localization of HydA-S is plastidial across the vast majority of taxa and has been experimentally confirmed in *C. reinhardtii* [87]*.* HydA-L is often predicted as mitochondrial.

**Fig. 8:**
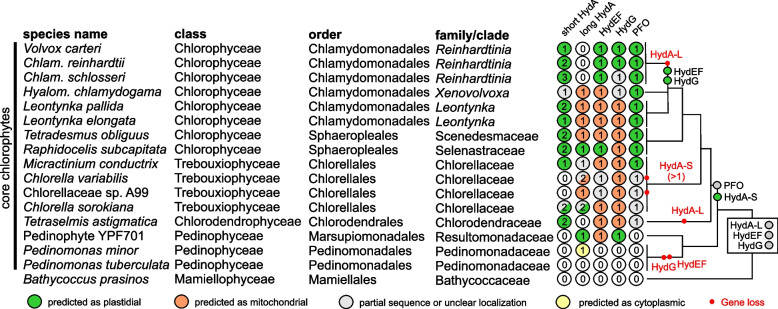
Occurrence of selected anaerobiosis-related proteins in core chlorophytes. The scheme displays the presence and the number of variants of FeFe hydrogenase (HydA, short and long version), its maturases (HydEF and HydG), and pyruvate:ferredoxin oxidoreductase (PFO) in selected representatives of core chlorophytes with high-quality genomes/transcriptomes available. The major evolutionary events shaping the complement of these enzymes in different lineages are indicated on the left, mapped onto a schematic phylogeny of the group. The results of in silico predictions of protein localizations are provided in Additional file 2: Table S15

The proper assembly of the hydrogenase H-cluster generally relies on three maturases, HydE, HydF, and HydG, with the first two fused in *C. reinhardtii*, forming the HydEF protein. Regardless of the number and the type of *hydA* genes present in the genome, core chlorophytes typically contain only one set of hydrogenase maturases (see Additional file 1: Figs S20 and S21). Both maturases are generally predicted as mitochondrial in core chlorophytes including *Leontynka* spp., which agrees with the prevalent mitochondrial localization of the canonical HydA-L (Fig. 8). If the hydrogenase maturases are truly missing from the *Leontynka* plastid, how the maturation of its plastidial HydA-S is secured remains unclear. In contrast, the ancestor of the *Reinhardtinia* phylogroup of Chlamydomonadales, which also includes *C. reinhardtii*, lost the mitochondrial HydA-L and relocalized all maturases to the plastid where both its HydA-S copies are also localized.

Acetyl-CoA is a precursor for the various anabolic and catabolic pathways [88], and it can also be further utilized for anaerobic ATP production. *Leontynka* spp. contain ATP forming/utilizing acetate kinase (ACK) and phosphate acetyltransferase (PAT) that can produce acetyl-CoA from acetate. This route is reversible in *C. reinhardtii*, where it generates ATP in anoxic conditions and produces acetate as a waste product, whereas it mediates acetate assimilation at normoxia [89]. *L. pallida* contains multiple homologs of ACK and PAT, while *L. elongata* possesses only one copy of each. One PAT homolog is predicted as plastidial in each species. In *L. elongata*, the localization prediction for ACK is inconclusive. In *L. pallida*, one ACK is most likely mitochondrial, as indicated by automatic prediction and confirmed by the manual inspection of its N-terminus (see Additional file 2: Table S16). Localization of the other ACK/PAT homologs is unclear, since the available sequences are truncated at the N-terminus or the prediction of their localization is inconclusive. Because PAT without ACK in the same compartment makes little physiological sense, our null hypothesis is that the ACK/PAT pathway is localized in the plastid of *L. elongata*, while *L. pallida* exhibits at least one plastidial and one mitochondrial copy of the pathway.

Our phylogenetic analyses revealed that the ACK and PAT enzymes originated deep in the evolution of green algae. Genes for both enzymes are present in both major lineages of Chloroplastida, namely Chlorophyta and Streptophyta. Moreover, they form a well-supported clade in the global phylogenetic tree of the respective enzymes (see Additional file 1: Figs S22 and S23). This strongly indicates that the ACK/PAT pathway was present in the ancestor of Chloroplastida. Both genes then underwent independent duplications. Chlamydomonadales of the *Reinhardtinia* phylogroup possess two distant ACK and PAT copies that probably originated by gene duplication early in the *Reinhardtinia* evolution. The two copies in *Reinhardtinia* are functionally diversified, as seen in *C. reinhardtii*, where one pair is plastidial and the other mitochondrial.

## Discussion

### Adaptations of *Leontynka* to low oxygen

*Leontynka* is a genus of non-model organisms that were isolated from microoxic habitats [13]. While our growth experiments clearly showed that *Leontynka* thrives in microoxic conditions (Fig. 1), both species possess classical aerobic mitochondria with functional Krebs cycle and OXPHOS. Furthermore, our analyses of the transcriptome data from both *Leontynka* spp. indicated that they do not possess many of the metabolic adaptations found in typical eukaryotic anaerobes. For instance, we did not find the rhodoquinone biosynthesis-specific methyltransferase RquA that is responsible for the synthesis of rhodoquinone, an alternative to ubiquinone that allows for utilizing fumarate as the terminal electron acceptor of the respiratory chain [90]. Some eukaryotic anaerobes can also form ATP from arginine using the arginine deiminase (ADI) pathway [91], but it is incomplete and hence likely non-functional in *Leontynka* due to the lack of carbamate kinase (see Additional file 2: Tables S9 and S10). This enzyme is also missing from most members of Chlorophyta, indicating that the remaining two enzymes operate outside of the ADI pathway. The transcriptome data also indicate that *Leontynka* does not possess the enzymes that extend the standard glycolytic pathway in typical eukaryotic anaerobes to generate further ATP by substrate phosphorylation, namely acetate:succinate CoA transferase (ASCT) and ADP-forming acetyl-CoA synthetase (ACS) [92].

**Fig. 9:**
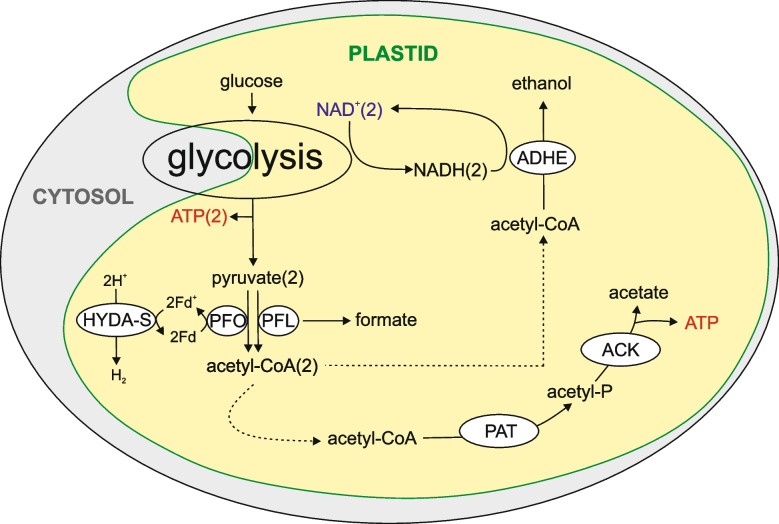
The plastid as the hub of the anaerobic energy metabolism in *Leontynka*. This scheme provides a simplified overview of the key processes of the anaerobic energy metabolism inferred to occur in the *Leontynka* plastid based on the data and analyses presented in this paper. The meaning of the abbreviations denoting the different enzymes is the same as explained in the legend to Fig. 5. The numbers at the compounds indicate the stoichiometry of the process (no number indicated implies a single molecule)

Instead, we suggest that *Leontynka* benefited from a versatile metabolism of its ancestors and has refined it to emphasize pathways essential for anaerobic metabolism within its plastid (Fig. 9). One the obvious adaptations of *Leontynka* that facilitate its survival in prolonged anoxia (or hypoxia) is accumulation of various energy storage compounds such as starch and polyphosphate in its plastid. Both compounds are present in a wide range of core chlorophytes [47, 93]. Catabolic metabolism of these compounds can compensate, at least temporarily, for the lower efficiency of ATP production when O_2_ cannot be used as a terminal electron acceptor in the mitochondrial OXPHOS. Indeed, our observations indicate that *Leontynka* consumes its starch reserves under anoxic conditions. This likely reflects the breakdown of starch into glucose that feeds into the plastid-localized upper part of the glycolytic pathway. Our in silico reconstruction of the* Leontynka* metabolic network (Fig. 5) indicates that the end product of glycolysis, i.e. pyruvate, can be further processed in along different routes depending on the conditions, with one of the routes constituting a variant of extended glycolysis allowing for extra ATP production even when pyruvate cannot be metabolized aerobically.

Specifically, under sufficient O_2_ supply pyruvate is converted in *Leontynka* (like in standard aerobic eukaryotes) to acetyl-CoA by the pyruvate dehydrogenase complex (PDH). As is common in plants and algae, *Leontynka* has a mitochondrial and a plastid version (mtPDH and ptPDH, respectively), yielding acetyl-CoA that is fed into the Krebs cycle and fuels fatty acid synthesis, respectively [94]. NADH generated in the mtPDH reaction is then reoxidized by the mitochondrial respiration chain, whereas in plastids, NADH reoxidation is mediated by a plastid-targeted type II NADH dehydrogenase (NDH2, Fig. 5). When oxygen becomes limited, *Leontynka* may regenerate NAD^+^, consumed in glycolysis, by reducing pyruvate to lactate. Crucially, it may alternatively use PFO or PFL to anaerobically (without NADH formation) convert pyruvate to acetyl-CoA and then reduce the latter in two consecutive ADHE-catalyzed reactions to produce ethanol, with each step using electrons from NADH [95]. Two NAD^+^ molecules per a single pyruvate molecule are thus regenerated by the ADHE-mediated pathway, allowing the second pyruvate molecule generated by the breakdown of a glucose molecule to enter the extended glycolytic pathway. We therefore propose that ethanol fermentation via ADHE constitutes the primary mechanism for NAD⁺ recycling in the *Leontynka* plastid under low-oxygen conditions.

According to our bioinformatic reconstructions, the extended glycolysis in *Leontynka* is localized into the plastid and instead of proceeding via the common ASCT/ACS pathway it yields ATP by employing acetate kinase (ACK) and phosphate acetyltransferase (PAT), two enzymes that our analyses show to have been acquired early in Chloroplastida evolution. This alternative ACK/PAT pathway is composed of two reversible reactions that can either mediate assimilation of external acetate by converting it to acetyl-CoA that is subsequently utilized by various catabolic and anabolic pathways, or uses acetyl-CoA to generate ATP anaerobically with acetate as a waste product. Indeed, operation of the pathway in the direction from acetyl-CoA to acetate has been directly demonstrated in plastids of *C. reinhardtii* grown in dark anoxia [89].

### Does *Azospirillum* hold a key to why *Leontynka* prefers microoxic conditions?

Although *Leontynka* retains some rare ancestral homologs of anaerobic metabolism enzymes, we did not identify in it any novel newly obtained enzyme that would explain its surprising affinity to (and better growth in) low oxygen. The mitochondria of *L. elongata* are localized close to the cell surface (see Additional file 1: Fig. S15), possibly to facilitate access to O_2_ in the hypoxic/microoxic environment. Since *Leontynka* spp. are osmotrophs, their affinity to microoxic environments cannot be explained by a higher abundance of prey bacteria. Still, metabolic connections of *Leontynka* to bacteria are very likely, as syntrophic interactions of free-living protists with bacteria in anoxic or hypoxic settings seem to be a common phenomenon (e.g., [96] and references therein). Indeed, both *Leontynka* species have been growing in the laboratory for more than 10 years together with co-isolated prokaryotes and our attempts to establish an axenic culture for *Leontynka* spp. were unsuccessful. This poses significant limitations for experimental work yet on the other hand, we can study the *Leontynka* metabolism in the context of prokaryotes that most likely were part of the original microbial community from which our cultures are derived. We noticed that both *Leontynka* cultures, established from sediments isolated more than 1000 km apart, share seven lineages of bacteria, anaerobic or microaerophilic. These include nitrogen-fixing *Azospirillum*, a bacterium known to promote plant and microalgal growth [97]. Specifically, *Azospirillum* produces phytohormones and improves the physiological activity of the green alga *Chlorella* when co-cultured with it [98, 99]. When tested on other core chlorophytes such as *Chlamydomonas* and *Scenedesmus*, *Azospirillum* showed beneficial effects on their growth as well [100].

While these interactions are synthetic, the interactions between *Leontynka* and *Azospirillum* are most likely natural. Therefore, they could be results of fine-tuned co-evolution. A close cooperation between *Leontynka* and microaerophilic *Azospirillum* or the other six anaerobic lineages might be the best explanation of *Leontynka*’s increased growth in microoxic conditions. We plan to elaborate on this interaction in the future. Why do some other chlorophytes (e.g., *C. reinhardtii*) still encode the same set of anaerobic enzymes and remain capable of surviving in low oxygen? The simplest explanation is that this capability helps them endure complex natural environments where O_2_ levels fluctuate. However, engaging in beneficial interactions with microaerophilic or anaerobic bacteria, such as *Azospirillum*, in their natural habitats, as seen in *Leontynka*, might be another reason. It was proposed that symbiotic anaerobic prokaryotes play a key role in the shift towards an obligate anaerobic lifestyle by clearing excess metabolic byproducts (such as H_2_), enhancing the overall metabolic rate, and generating sufficient energy to allow aerobic pathways to be occasionally lost [101]. If our hypothesis is correct, possible drivers of the same process in Chlamydomonadales are free-living anaerobic or microaerophilic bacteria such as *Azospirillum*.

### Metabolic similarities between the plastid of *Leontynka* and a hydrogenosome

The Chlamydomonadales ancestor was a photosynthetic organism that produced O_2_ and organic compounds by autotrophy, but was also capable of heterotrophic growth by exploiting external sources of energy and carbon, which included assimilation of acetate by a plastid-localized ACK/PAT pathway. However, the latter process could function in reverse when external acetate concentration remained sufficiently low and was thus used for anaerobic ATP generation under anoxia. This ancestral metabolic versatility is largely retained in *C. reinhardtii*, although some enzymes have relocated to different cellular compartments compared to the ancestral state (e.g., hydrogenase maturases). In contrast, *Leontynka* spp. lost their photosynthetic machinery and have become obligately heterotrophic. However, the *Leontynka* plastid still retains a number of metabolic functions. The plastid-localized steps of glycolysis are of particular interest for this study. The upper part of glycolysis is performed in the plastid, while only the later reactions are cytosolic. However, the very final step, i.e., the ATP generation by PEP-to-pyruvate conversion, occurs also in the plastid in parallel to the cytosol in *Leontynka* spp. (Fig. 3). Under normoxia, when the highly efficient mitochondrial OXPHOS metabolism is operational as the key source of ATP for the cell, the ATP production catalyzed by plastid-localized paralogs of PK may primarily serve the needs of the metabolic processes in the *Leontynka* plastid itself. However, we posit that in the shortage of O_2_ the plastid becomes the hub of the energy metabolism, producing ATP by an extended glycolysis producing a mixture of ethanol and acetate, possibly also formate depending on the relative contribution of PFO and PFL to the pyruvate conversion to acetyl CoA (Fig. 9). The function of PFO critically depends on ferredoxin reoxidation by FeFe hydrogenase, and hence H_2_ release by the *Leontynka* plastid is predicted to occur. Although we do not have direct experimental evidence for the proposed metabolic model, it seems to naturally emerge from our bioinformatic analyses and is generally consistent with previous studies of anaerobic metabolism in *C. reinhardtii*.

## Conclusions

Here, we employed the non-photosynthetic *genus Leontynka* as a model to investigate the evolution of anaerobiosis in Chlamydomonadales and characterized its energy metabolism. This work provides the first in-depth examination of microaerophilic algae within this order and places their metabolic capabilities into a context through comparison with *Chlamydomonas reinhardtii* and *Polytomella parva*. It is interesting to note that by having lost its main conventional function, i.e. photosynthesis, and assuming the central role in anaerobic energy metabolism, the *Leontynka* plastid is reminiscent of modified forms of the mitochondria found in various obligate anaerobic or microaerophilic eukaryotes, namely hydrogenosomes or the so-called hydrogen-producing mitochondria [102]. Besides making ATP by substrate-level phosphorylation in an extended glycolysis and releasing H_2_, the parallels between the *Leontynka* plastid and hydrogenosomes lie in the loss of membrane complexes that exploit redox reactions to build a proton gradient between the intermembrane space and the stroma/matrix, i.e. the photosynthetic electron transport chain and the respiratory chain, respectively. This feature represents a distinctive characteristic of the *Leontynka* plastid that is not shared with *Chlamydomonas reinhardtii* and other photosynthetic members of Chlamydomonadales. Our study thus further underscores the enormous metabolic diversity of non-photosynthetic plastids [49, 50, 103] and points to an interesting case of convergence in the evolution of endosymbiotic organelles.

## Methods

### Culturing, growth experiments, and light microscopy

*L. elongata* (strain MBURUCU) and *L. pallida* (strain AMAZONIE) were maintained in polyxenic agnothobiotic cultures grown on the ATCC medium 802 at room temperature in darkness (the pH of the medium was not adjusted). Cultures of *L. pallida* used for growth experiments were established from a single culture passage grown for 2 weeks in 50 ml of the medium. Details of the experimental design for growth experiments are described in Additional file 3: Methods S1 and Additional file 1: Fig. S1. To observe starch granules, cells were stained with 50% Lugol solution and were immediately observed under a BX51 microscope (Olympus).

### Culturing, RNA extraction, transcriptome sequencing, and assembly

One hundred milliliters of *L. elongata* culture was used for total RNA extraction using the TRIzol Reagent (Invitrogen) following the manufacturer’s extraction protocol. The (meta)transcriptome was sequenced with NovaSeq 6000 (2 × 150 bp) paired-end technology, with libraries prepared using the TruSeq stranded mRNA workflow at Macrogen Inc. (South Korea). Transcriptome assembly was done with Trinity 2.9.1 (slidingwindow:4:5 leading:5 trailing:5 minlength:50) [104]. Coding regions were predicted using TransDecoder v5.5.0 (-m 80) [104] and CD-HIT v4.8.1 (-c 0.99) [105] was used for sequence clustering. A total of 49,770,850 reads were assembled into 88,754 contigs. The same methodology was used to assemble publicly available reads from Chlamydomonadales sp. NrCl902 and predict its proteome. The completeness of the predicted proteomes used in the study was assessed using BUSCO v5 [106] in the protein mode. To assess the taxonomic composition of the microbial community sequenced together with the target *Leontynka* species, prokaryotic SSU rRNA gene sequences were reconstructed using phyloFlash [107] (see Additional file 2: Tables S1 and S2).

### Trophic mode analysis

The predicted proteomes of *Leontynka* spp. together with proteomes of representative species of Chlamydomonadales available in the EukProt v3 database [108] were run in a default mode against the profile hidden Markov models of 14,095 protein clusters from 35 eukaryotes provided by the predictTrophicMode tool [33]. Significant hits were filtered to remove contaminants using BLASTp. Sequences with best hits corresponding to bacterial proteins were omitted; the remaining set was used in the downstream analysis of the predictTrophicMode tool. The final figure was generated using the R script provided by the authors [33].

### Decontamination and ortholog selection

Predicted proteomes from the newly generated *L. elongata* transcriptome, previously published transcriptome assemblies of *L. pallida* [37] and *Polytomella parva* SAG63 [36], and the newest *C. reinhardtii* genome assembly v6.1 [109] were searched with BLASTp for enzymes of interest. In total, we searched for homologs of 326 proteins involved in energy metabolism and other selected pathways of *C. reinhardtii*. The respective protein sequence queries were extracted from the annotated *C. reinhardtii* mitochondrial and plastid proteomes [14, 15] and published studies on the *C. reinhardtii* metabolism. We further enriched the query dataset using the experimentally defined plastid proteome from *Polytomella* spp. [74, 110] and queries representing several other anaerobiosis-associated enzymes not present in *C. reinhardtii* (e.g., RquA and ASCT). Potential homologs of target proteins identified in predicted proteomes through a BLASTp search were screened by comparison with the NCBI non-redundant (nr) database. Hits showing high sequence identity with prokaryotic proteins were marked as such. Protein annotations were evaluated using the KEGG genes/pathway database. In ambiguous cases, we performed phylogenetic analyses.

### Phylogenetic analyses

The same query sequences used in the identification of *Leontynka* orthologs were used as BLASTp queries at the MPI Bioinformatics Toolkit [111] to search separately for eukaryotic, bacterial, and archaeal protein sequences from the NCBI nr database filtered for maximum 70% pairwise identity (the latest precomputed versions of the nr_arc70, nr_bac70, and nr_euk70 databases available for searching at the server). Additional sequence data were collected from EukProt v3 database to identify contaminations and to expand sampling of Chlorophyta beyond the initial search. Protein sequences were aligned using MAFFT v7.453 [112] under the following settings: L-INS-i algorithm, gap opening penalty 1.3, leave gappy regions, and the BLOSUM30 scoring matrix. Alignments were trimmed either manually or using BMGE v1.12 (-g 0.9) [113]. Phylogenetic analyses were performed with IQ-TREE v2.0.3 [114] using the LG4X model (selected arbitrarily as a reasonably complex model, given the size of the matrices fed into the phylogenetic analyses), if not stated otherwise in the legend to the particular tree. Trees were annotated and exported as figures using ITOL [115]. All single-gene datasets and complete phylogenetic trees are deposited on Figshare [116].

### Prediction of protein subcellular localization

Subcellular localization of studied proteins was predicted by TargetP-2.0 [19], DeepLoc2.0 and DeepLoc2.1 [18], and PredAlgo [17] using the respective web interfaces. The latter tool was not used for final analyses due to the high percentage of false positives for mitochondrial proteins (see Results). We used default settings with the following options: Plant (TargetP-2.0) and High Quality (DeepLoc2.1). For details regarding final protein localization assignment and prediction software performance, see Additional file 3: Notes S2. We also compared characteristics of N-terminal sequences of *C. reinhardtii* and *Leontynka* spp. proteins to confirm the reliability of the automatic predictions.

### Raman microspectroscopy

Cells of *L. pallida* were harvested 36 h and 7 days after inoculation, respectively; cells of *L. elongata* 36 h after inoculation. The cells were centrifuged at 2500 × *g* for 3 min. The pellet was transferred onto a quartz microscopy slide and mixed with 2% low-temperature-melting agarose to immobilize the live cells. Pure chemical standards purchased from Sigma (Merck) were prepared similarly. Raman hyperspectral imaging was performed using a Raman microscope Witec alpha 300 RSA (Witec) equipped with a 60 × UPlanSApo, NA 1.2, water immersion objective (Olympus). Detailed settings and image processing are described in Additional file 2: Methods S2.

### Staining techniques and polarization microscopy

Fluorescence staining was performed on cells harvested 7 days after inoculation of *L. elongata* culture. Staining incubation period was 10 min. For staining, DAPI (4',6-diamidino-2-phenylindole, dilactate; Invitrogen, Carlsbad, CA, USA) was used at a final concentration of 1 μg/mL to stain DNA of the nucleus and mitochondria in permeabilized cells (using 1% TritonX), with excitation/emission maxima of 358/461 nm. Nile red (Carl Roth GmbH, Karlsruhe, Germany) dissolved in acetone was used at a final concentration of 1 μg/mL to stain lipid droplets, with excitation/emission maxima of 552/636 nm. SYTO13 (Molecular Probes, Eugene, OR, USA) was used at a concentration of 1 μM for live DNA/RNA staining of the nucleus, mitochondria, and plastids, with excitation/emission maxima of 488/509 nm. Imaging was done using an Olympus Provis AX70 microscope equipped with fluorescence filter cubes (U-MWU, U-MWIB, U-MWG) and a Nikon D3100 DSLR camera.

### High-performance liquid chromatography (HPLC)

Cells from 50–100-ml cultures were harvested by centrifugation (3500 × *g* at 4 °C for 5 min). The pigments from the pellets were extracted and analyzed by HPLC. For analysis of pigments from prokaryotes present in the *L. pallida* cultures, the prokaryote-enriched supernatant was centrifuged at 31,000 × *g* and 4 °C for 5 min. The pigments from the pellet were extracted and analyzed by HPLC in the same way. Pigments from the culture medium were extracted by thorough mixing of 5 ml of fresh medium with 2 ml of acetone and 2 ml of ethyl acetate and addition of 4 ml of NaCl solution (5 mol/L) to induce phase separation. The slightly yellowish epiphase was collected and dried by flushing with nitrogen gas. The dried pigments were redissolved in 200 μL of methanol and 200 μL of acetone and subjected to HPLC analysis as previously described by Blatt et al. [117]. Pigments were identified by comparison of retention times and absorbance spectra with those of reference pigments that were isolated from *Escherichia coli* strains engineered to synthesize different carotenoids. The zeaxanthin standard was prepared from *E. coli* TOP10 transformed with plasmid pACCARD25crtX [118], isomers of β-carotene or lycopene from TOP10 transformed with plasmids pBETA2 or pLYCO2, respectively [117], and γ-carotene isomers from TOP10 transformed with plasmid pAC-GAMMAipi [119]. When grown in liquid culture at 28 °C in darkness [117], the transgenic strains accumulated mostly the all-trans isomer of the respective carotenoid, together with small amounts of various *cis*-isomers of the main carotenoid likely resulting from thermal isomerization.

### Electron microscopy

Pelleted cells were high-pressure frozen (EM ICE, Leica) in the presence of 20% BSA and were freeze-substituted in 2% OsO_4_ in 100% acetone for 96 h at − 90 °C. The temperature was raised (5 °C/h) to − 20 °C and after 24 h raised again (4 °C/h) to 4 °C. At room temperature, the pellet was washed three times in 100% acetone and infiltrated in 25, 50, and 75% resin EMBed 812 diluted in acetone for 1 h at each step. After overnight incubation in pure resin, the samples were embedded and polymerized at 60 °C for 48 h. Ultrathin sections were stained with ethanolic uranyl acetate and lead citrate and were carbon-coated. Samples were examined by a 120-kV TEM JEM 1400 Flash (JEOL) equipped with a CMOS camera Xarosa (EMSIS), where regions of interest for tomography were identified. Data for two serial electron tomograms were collected using a 200-kV TEM JEM 2100 F (JEOL) equipped with a direct electron detector K2 Summit (Gatan). Each serial tomogram was generated by joining four dual-axis electron tomograms acquired from four consecutive sections. The individual tilt series was performed within a range of ± 60° with a tilt step of 1°. Tomograms were reconstructed and joined using the IMOD program package [120], where 3D models were created and cristae measurements were performed [121].

## Supplementary Information

Additional file 1: Fig. S1 Experimental set-up of the growth experiment under room temperature in darkness. Fig. S2 Comparison of prokaryotic communities in *Leontynka* cultures. Fig. S3 Assessment of the completeness of transcriptome-derived protein of *Leontynka* spp. Fig. S4 Charge and binding sites of predicted *Leontynka* transit peptides match expectations. Fig. S5 Key amino acids of predicted *Leontynka* transit peptides match expectations. Fig. S6 Phylogenetic tree of endo/exo-cellulases. Fig. S7 Multiple sequence alignment of β-glucosidase. Fig. S8. Components of *Leontynka elongata* cells visualized by a wide range of staining techniques and polarization microscopy. Fig. S9 Reconstruction of fatty acid synthesis and degradation in *Leontynka*. Fig. S10 HPLC analyses of pigment extracts from a *L. pallida* culture medium. Fig. S11 Carotenoid synthesis in *Leontynka*. Fig. S12 Multiple sequence alignment of β-carotene ketolase. Fig. S13 Phylogenetic tree of fructose-1,6-bisphosphatase class 2 (GlpX). Fig. S14 Cutouts from tomograms showing cristae in detail with cristae junctions at the center. Fig. S15 Position of mitochondria in *Leontynka elongata* cells. Fig. S16 Global phylogenetic tree of pyruvate formate lyase (PFL). Fig. S17 Phylogenetic tree of pyruvate:ferredoxin oxidoreductase (PFO). Fig. S18 Focused phylogenetic analysis of pyruvate formate lyase (PFL). Fig. S19 Phylogenetic tree of ferredoxins from Chloroplastida. Fig. S20 Phylogenetic tree of the fused hydrogenase maturase protein HydEF. Fig. S21 Phylogenetic tree of the hydrogenase maturase protein HydG. Fig. S22 Focused phylogenetic analysis of acetate kinase (ACK). Fig. S23 Focused phylogenetic analysis of phosphate acetyltransferase (PAT).

Additional file 2: Table S1 SSU rRNA of prokaryotes identified in *Leontynka* metatranscriptomes using PhyloFlash. Table S2 Bacterial contaminants identified in *Leontynka* metatranscriptomes. Table S3 Proteins from *Chlamydomonas reinhardtii* used to test the automatic prediction tools. Table S4 Accuracy of selected predictors as tested on mitochondrial proteome of *C. reinhardtii*. Table S5 Accuracy of selected predictors as tested on plastid proteome of *C. reinhardtii*. Table S6 Accuracy of selected predictors as tested on proteins that were experimentally localized in the mitochondrion of *C. reinhardtii*. Table S7 Accuracy of selected predictors as tested on proteins that were experimentally localized in the plastid of *C. reinhardtii*. Table S8 Functional annotations, amino acid sequences, and predicted localization of *Leontynka pallida* proteins investigated in this study. Table S9 Functional annotation, amino acid sequences, and predicted localization of *Leontynka elongata* proteins investigated in this study. Table S10 Functional annotation, amino acid sequences, and predicted localization of *C. reinhardtii* proteins investigated in this study. Table S11 Functional annotation, amino acid sequences, and predicted localization of *Polytomella parva* proteins investigated in this study. Table S12 General features of cells used to define position of mitochondria. Table S13 Source data for Supporting Figure S14. Measurements are listed in % and px. Table S14 Functional annotation, predicted localization, and presence of F-cluster in FeFe hydrogenases. Table S15 Functional annotation, amino acid sequences, and predicted localization of PFO, FeFe hydrogenase, its maturases, and PFO in Chlamydomonadales. Table S16 Source data for Additional file 1: Figs. S3 and S4, and manual examination of typical features of plastid transit peptides or mitochondrial transit peptides in selected proteins. Table S17 Functional annotation, amino acid sequences, and predicted localization of Chlamydomonadales strain NrCl902 investigated in this study.

Additional file 3: Methods S1 Details of growth experiments. Methods S2 Raman microscopy (settings and image processing). Notes S1 Evaluation of transcriptome completeness. Notes S2 Evaluation of protein localization predictions. Notes S3 Cellulose degradation pathway. Notes S4 Cristae and cristae junctions of *Leontynka elongata*.

Additional file 4: Raman microscopy of *Leontynka pallida* and *Leontynka elongata* (video).

## Data Availability

The data that supports the findings of this study are available in the supplementary material of this article. Datasets and raw outputs corresponding to phylogenetic analyses and unfiltered transcriptome assembly of *Leontynka elongata* are openly available in the Figshare repository [116]. Raw transcriptomic reads of *Leontynka elongata* have been deposited at the NCBI SRA database under the accession SRR33614310 [122].

## Abbreviations

TEM: Transmission electron microscopy
CBB: Calvin–Benson–Bassham cycle
cTP: Chloroplast transit peptides
mTP: Mitochondrial transit peptides
VTC4: Vacuolar transporter chaperone complex subunit 4
TAGs: Triacylglycerols
MEP pathway: Methyl-D-erythritol 4-phosphate pathway
IPP: Isopentenyl pyrophosphate
HPLC: High-performance liquid chromatography
CHYB: β-Carotene hydroxylase
CRTISO: Carotenoid isomerase
BKT: β-Carotene ketolase
CYP51: Obtusifoliol 14α-demethylase
SMO: Sterol-C₄-methyl oxidase
CYP710: Sterol C-22 desaturase
STE1: Δ^7^-Sterol-C_5_-desaturase
STC: Squalene-tetrahymanol cyclase
SHC: Squalene–hopene cyclase
GAPDH: Glyceraldehyde-3-phosphate dehydrogenase
PTOX: Plastid terminal oxidase
PEP: Phosphoenolpyruvate
PPDK: Pyruvate, phosphate dikinase
PK: Pyruvate kinase
NADH: Nicotinamide adenine dinucleotide
PEPCK: Phosphoenolpyruvate carboxykinase
FBPaseI: Fructose-1,6-bisphosphatase class I
FBPaseII: Fructose-1,6-bisphosphatase class II
GlpX: Fructose-1,6-bisphosphatase class 2, GlpX-type
OXPHOS: Oxidative phosphorylation
CJ: Crista junction
ADHE: Aldehyde/alcohol dehydrogenase
PDC: Pyruvate decarboxylase
PFL: Pyruvate formate lyase
PFO: Pyruvate:ferredoxin oxidoreductase
ADH3: Cytosolic alcohol dehydrogenase
PFLA: Pyruvate formate lyase
FDX1, PETF: Ferredoxin 1
HydA-S: Short FeFe hydrogenase
HydA-L: Long FeFe hydrogenase
ACK: Acetate kinase
PAT: Phosphate acetyltransferase
ADI: Arginine deiminase
ASCT: Acetate:succinate CoA transferase
ACS: ADP-forming acetyl-CoA synthetase
PDH: Pyruvate dehydrogenase complex
NDH2: Type-II NADH dehydrogenase
PDH: Pyruvate dehydrogenase
HydG: Hydrogenase assembly factor G
HydEF: Hydrogenase maturase
