# Supplementary material for: Energy metabolism and adaptation to hypoxia in the non-photosynthetic green alga Leontynka
Source: BMC Biol. 2026 Jan 29;24:50. doi: 10.1186/s12915-026-02529-3 (PMC12924338; doi:10.1186/s12915-026-02529-3)
Supplement: Supplementary file 1 — Additional file 1: Fig. S1 Experimental set-up of the growth experiment under room temperature in darkness. Fig. S2 Comparison of prokaryotic communities in Leontynka cultures. Fig. S3 Assessment of the completeness of transcriptome-derived protein of Leontynka spp. Fig. S4 Charge and binding sites of predicted Leontynka transit peptides match expectations. Fig. S5 Key amino acids of predicted Leontynka transit peptides match expectations. Fig. S6 Phylogenetic tree of endo/exo-cellulases. Fig. S7 Multiple sequence alignment of β-glucosidase. Fig. S8. Components of Leontynka elongata cells visualized by a wide range of staining techniques and polarization microscopy. Fig. S9 Reconstruction of fatty acid synthesis and degradation in Leontynka. Fig. S10 HPLC analyses of pigment extracts from a L. pallida culture medium. Fig. S11 Carotenoid synthesis in Leontynka. Fig. S12 Multiple sequence alignment of β-carotene ketolase. Fig. S13 Phylogenetic tree of fructose-1,6-bisphosphatase class 2 (GlpX). Fig. S14 Cutouts from tomograms showing cristae in detail with cristae junctions at the center. Fig. S15 Position of mitochondria in Leontynka elongata cells. Fig. S16 Global phylogenetic tree of pyruvate formate lyase (PFL). Fig. S17 Phylogenetic tree of pyruvate:ferredoxin oxidoreductase (PFO). Fig. S18 Focused phylogenetic analysis of pyruvate formate lyase (PFL). Fig. S19 Phylogenetic tree of ferredoxins from Chloroplastida. Fig. S20 Phylogenetic tree of the fused hydrogenase maturase protein HydEF. Fig. S21 Phylogenetic tree of the hydrogenase maturase protein HydG. Fig. S22 Focused phylogenetic analysis of acetate kinase (ACK). Fig. S23 Focused phylogenetic analysis of phosphate acetyltransferase (PAT). [file 12915_2026_2529_MOESM1_ESM.docx]

**ADDITIONAL FILE 1: SUPPLEMENTARY FIGURES**

**Article title:** Energy metabolism and adaptation to hypoxia in the non-photosynthetic green alga *Leontynka*

**Authors:** Pia Corre, Jana Pilátová, Tomáš Bílý, Eliška Zadrobílková, Ivan Čepička, Marie Vancová, Martin Lohr, Oliver D. Caspari, Marek Eliáš, Tomáš Pánek

**Fig. S1** Experimental set-up of the growth experiment under room temperature in darkness.

**Fig. S2** Comparison of prokaryotic communities in *Leontynka* cultures.

**Fig. S3** Assessment of the completeness of transcriptome-derived protein of *Leontynka* spp.

**Fig. S4** Charge and binding sites of predicted *Leontynka* transit peptides match expectations.

**Fig. S5** Key amino acids of predicted *Leontynka* transit peptides match expectations.

**Fig. S6** Phylogenetic tree of endo/exo-cellulases.

**Fig. S7** Multiple sequence alignment of β-glucosidase.

**Fig. S8.** Components of *Leontynka elongata* cells visualized by a wide range of staining techniques and polarization microscopy.

**Fig. S9** Reconstruction of fatty acid synthesis and degradation in *Leontynka*.

**Fig. S10** HPLC analyses of pigment extracts from a *L. pallida* culture medium.

**Fig. S11** Carotenoid synthesis in *Leontynka*.

**Fig. S12** Multiple sequence alignment of β-carotene ketolase

**Fig. S13** Phylogenetic tree of fructose-1,6-bisphosphatase class 2 (GlpX).

**Fig. S14** Cutouts from tomograms showing cristae in detail with cristae junctions at the center.

**Fig. S15** Position of mitochondria in *Leontynka elongata* cells.

**Fig. S16** Global phylogenetic tree of pyruvate formate lyase (PFL).

**Fig. S17** Phygenetic tree of pyruvate:ferredoxin oxidoreductase (PFO).

**Fig. S18** Focused phylogenetic analysis of pyruvate formate lyase (PFL).

**Fig. S19** Phylogenetic tree of ferredoxins from Chloroplastida.

**Fig. S20** Phylogenetic tree of the fused hydrogenase maturase protein HydEF.

**Fig. S21** Phylogenetic tree of the hydrogenase maturase protein HydG.

**Fig. S22** Focused phylogenetic analysis of acetate kinase (ACK).

**Fig. S23** Focused phylogenetic analysis of phosphate acetyltransferase (PAT).


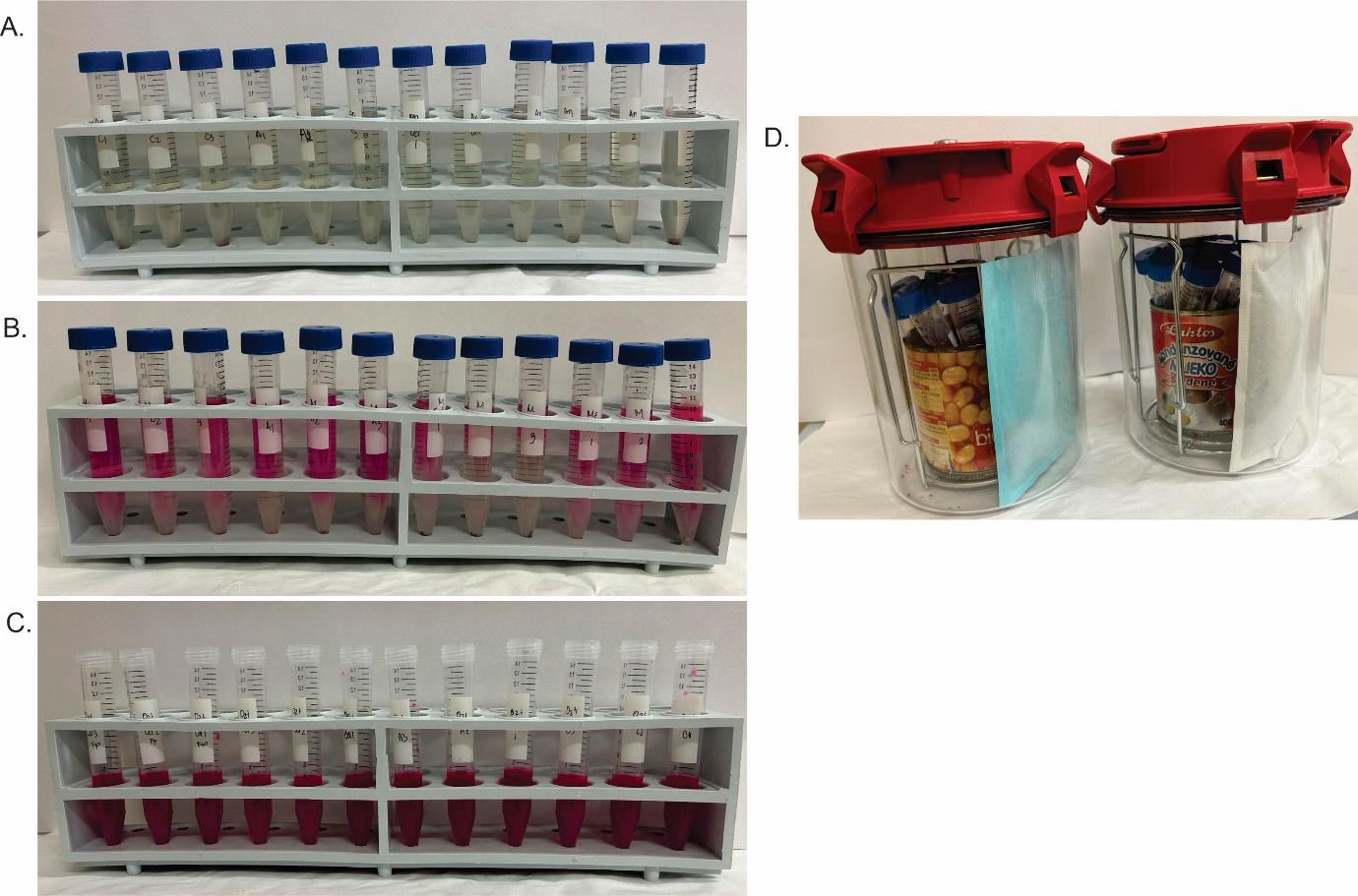


**Fig. S1. Experimental set-up of the growth experiment under room temperature in darkness.** **A.** Replicates grown in anoxia. **B.** Replicates grown in microoxic conditions. **C.** Replicates grown in normoxia (open tubes, less medium). All replicated contained resazurin dye to monitor oxygen levels (colorless when reduced; pink in presence of oxygen). **D.** Anaerojars with CampyGEN and AnaeroGEN gas generator for microoxic (left) and anoxic (right) conditions, respectively.


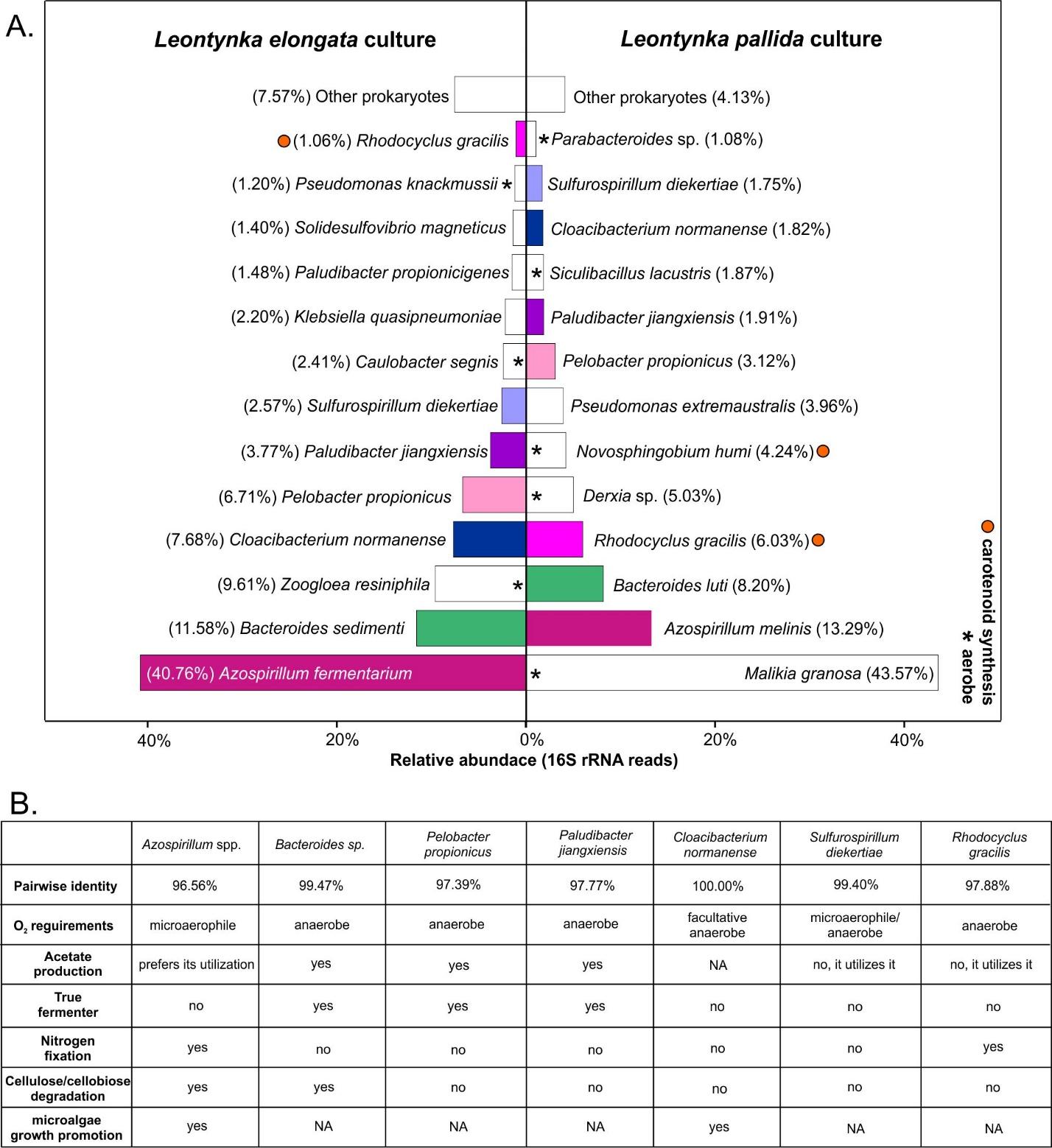


**Fig. S2.** **Comparison of prokaryotic communities in *Leontynka* cultures.** **A.** Relative abundance based on 16S rRNA gene reads contaminating transcriptomes (Illumina). Lineages with abundance < 1% are grouped into the category “other prokaryotes” as they likely represent contaminants. Strictly aerobic and carotenoid-producing lineages are indicated (see graphical legend). Closely-related lineages present in both cultures are indicated by colored bars. Source data are available as Additional file 2: Table **S1**. **B.** Analysis of overlapping bacterial community; it summarizes pairwise identity among strains from the *Leontynka* cultures and the basic metabolic traits of their closest characterized relatives. While the 16S rRNA sequence divergence between the two *Azospirillum* lineages indicates that they represent different species (*A. melinis* and *A. fermentarium*), the remaining lineages most likely represent very closely related species—or possibly even the same species—present in both cultures. Abbreviation: NA – not assigned.


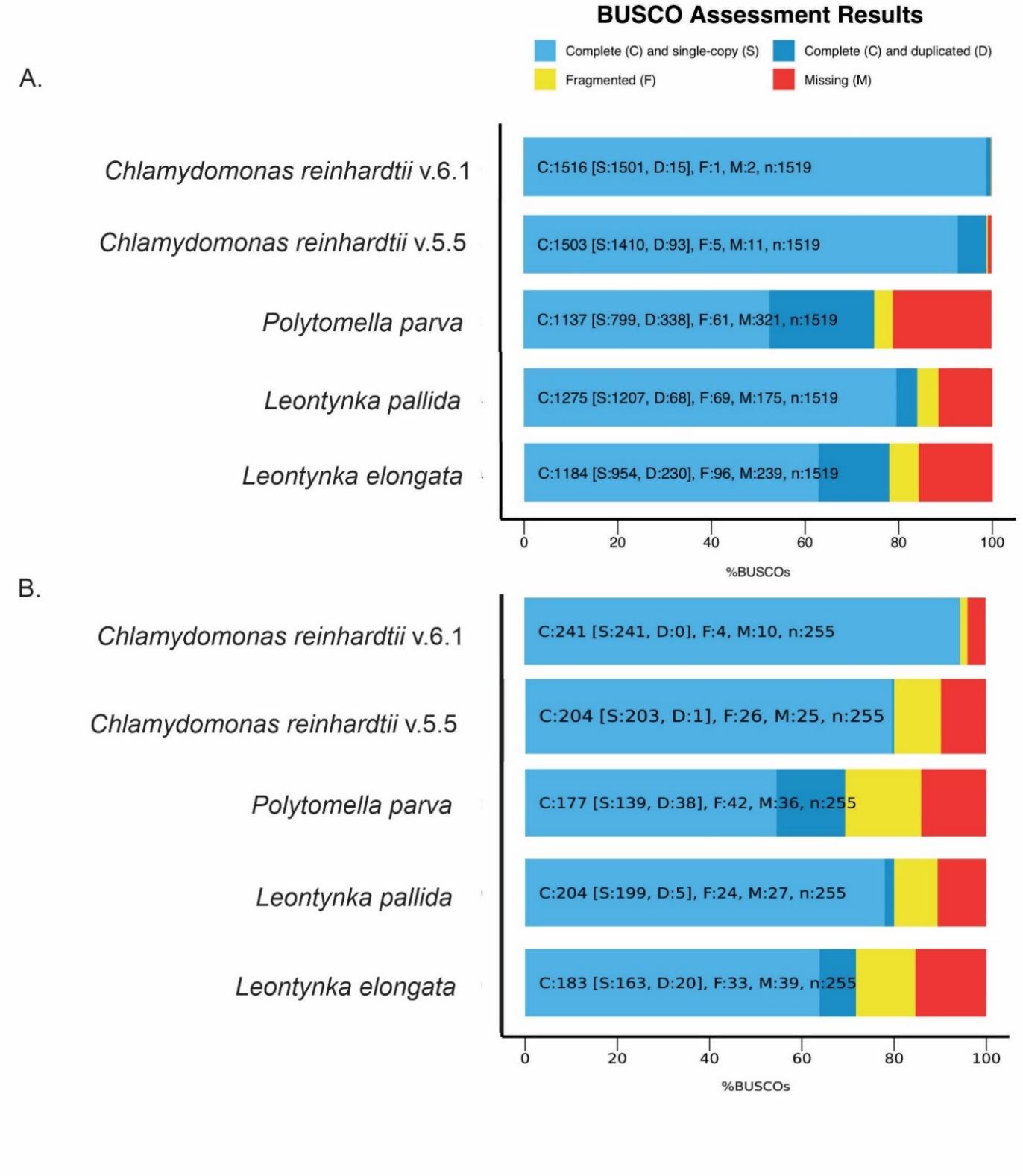


**Fig. S3. Assessment of the completeness of transcriptome-derived protein datasets of *Leontynka* spp.** Statistics are based on BUSCO v5 using **A.** chlorophyta_odb10 dataset, **B.** eukaryote_odb10 dataset. Sources of transcriptomic data used for the analysis are listed in Material and Methods. Predicted proteins from *Chlamydomonas reinhardtii* genome (v.5.5 and v.6.1) were used as a reference.

**
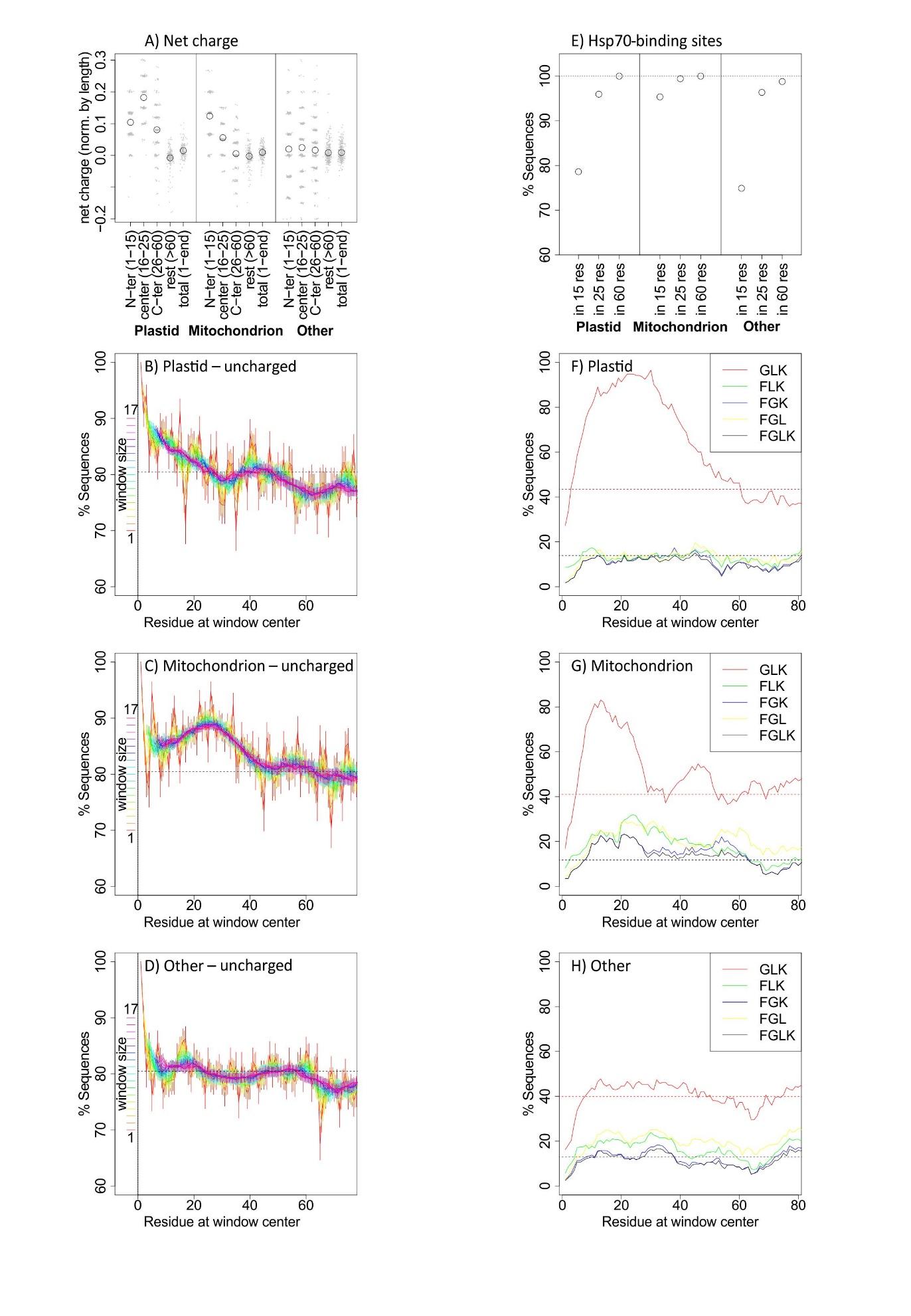
**

**Fig. S4.** **Charge and binding sites of predicted *Leontynka* organelle transit peptides match expectations.** Proteins predicted to target the plastid, the mitochondrion, or other/undetermined subcellular locations were treated as separate classes. **A.** Net charge (K/R counted as +, D/E as -) divided by sequence length. Protein sequences were divided into three sections at the N-terminus, reflecting the tripartite nature of plastid transit peptides. Residues 1-5 were taken as an N-terminal sequence, residues 16**–**25 as the transit peptide center, and residues 26**–**60 as a peptide C-terminus, before the rest of the protein. For comparison, the equivalent sections are shown for all predicted localization classes. Gray dots show individual sequences (with random noise added in x and y to visibly spread the points), black circles show average values. Both mitochondrial and chloroplast targeting sequences are known to carry net positive charges, whereas plastid transit peptides contain a relatively uncharged N-terminus. **B**–**D.** The proportion of uncharged residues across proteins is shown for varying sequences lengths (‘windows’ used for the analysis) from 5 residues (red) to 30 residues (purple), with the average across all sequences shown as the horizontal line. **B.** Predicted plastid sequences show a relatively uncharged N-terminus. **C.** Predicted mitochondrial sequences have a relatively uncharged stretch later in the sequence. **D.** Other sequences hover around the average across all sequences (dashed line). **E.** Predicted Hsp70-binding sites within the first 15/35/60 residues. **F–H.** ‘FGLK’-sites (semi-conserved putative TOC-binding sites) and sites with one fewer amino acid along the sequences. Horizontal lines show the average across sequences when the order of amino acids was randomized. **F.** Most predicted plastid sequences contain ‘FGLK’-sites within 60 residues, especially between residues 10-35. **G.** Predicted mitochondrial sequences often contain ‘(F)GLK’-sites within the first ca. 25 residues. **H.** Other sequences do not contain more sites than expected by chance.


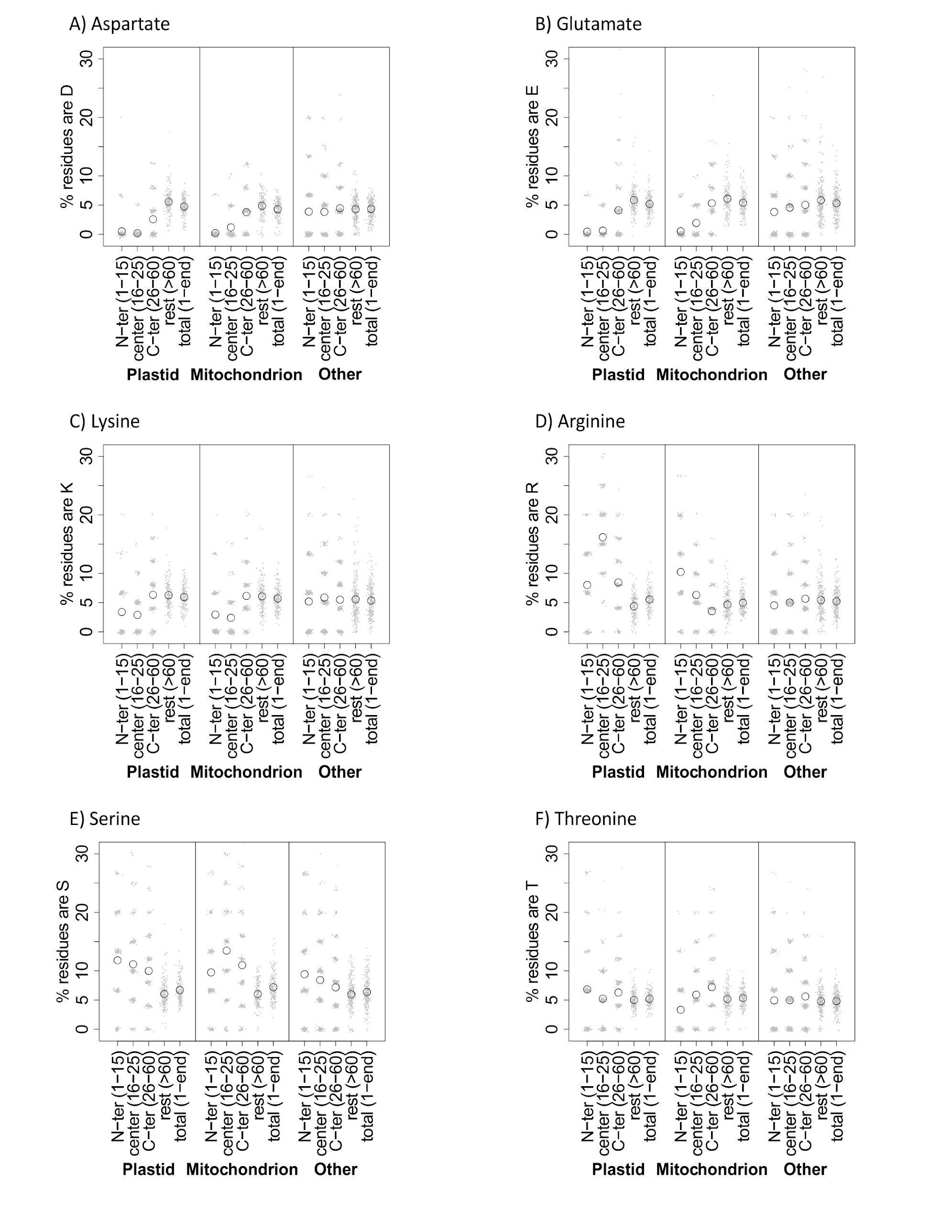


**Fig. S5.** **Key amino acids of predicted *Leontynka* organelle transit peptides match expectations.** Proteins predicted to target the plastid, the mitochondrion, or other/undetermined subcellular locations were treated as separate classes. Protein sequences were divided in three sections at the N-terminus, reflecting the tripartite nature of plastid transit peptides. Residues 1**–**5 were taken as N-terminal sequence, residues 16**–**25 as the transit peptide center, and residues 26**–**60 as peptide C-terminus, before the rest of the protein. For comparison, the equivalent sections are shown for all predicted localization classes. Gray dots show individual sequences (with random noise added in x and y to visibly spread the points), black circles show average values. **A.** Aspartate (D) and **B.** Glutamate (E) are noticeably absent from the N-termini of predicted plastid and mitochondrial sequences. **C.** Lysine (K) is also lower, but **D.** Arginine (R) is much higher than in the rest of the protein sequences or in proteins predicted to target other destinations. **E.** Phosphorylatable serine (S) is increased in N-termini generally, but in predicted plastid and mitochondrial sequences more so than in other sequences. **F.** The other phosphorylatable residue, threonine (T), is roughly present in the same proportion in N-terminus and later sequence parts across the board. Only in predicted mitochondrial sequences, both serine and threonine are noticeably lower at the very N-terminus compared to the next sequence stretch.

**
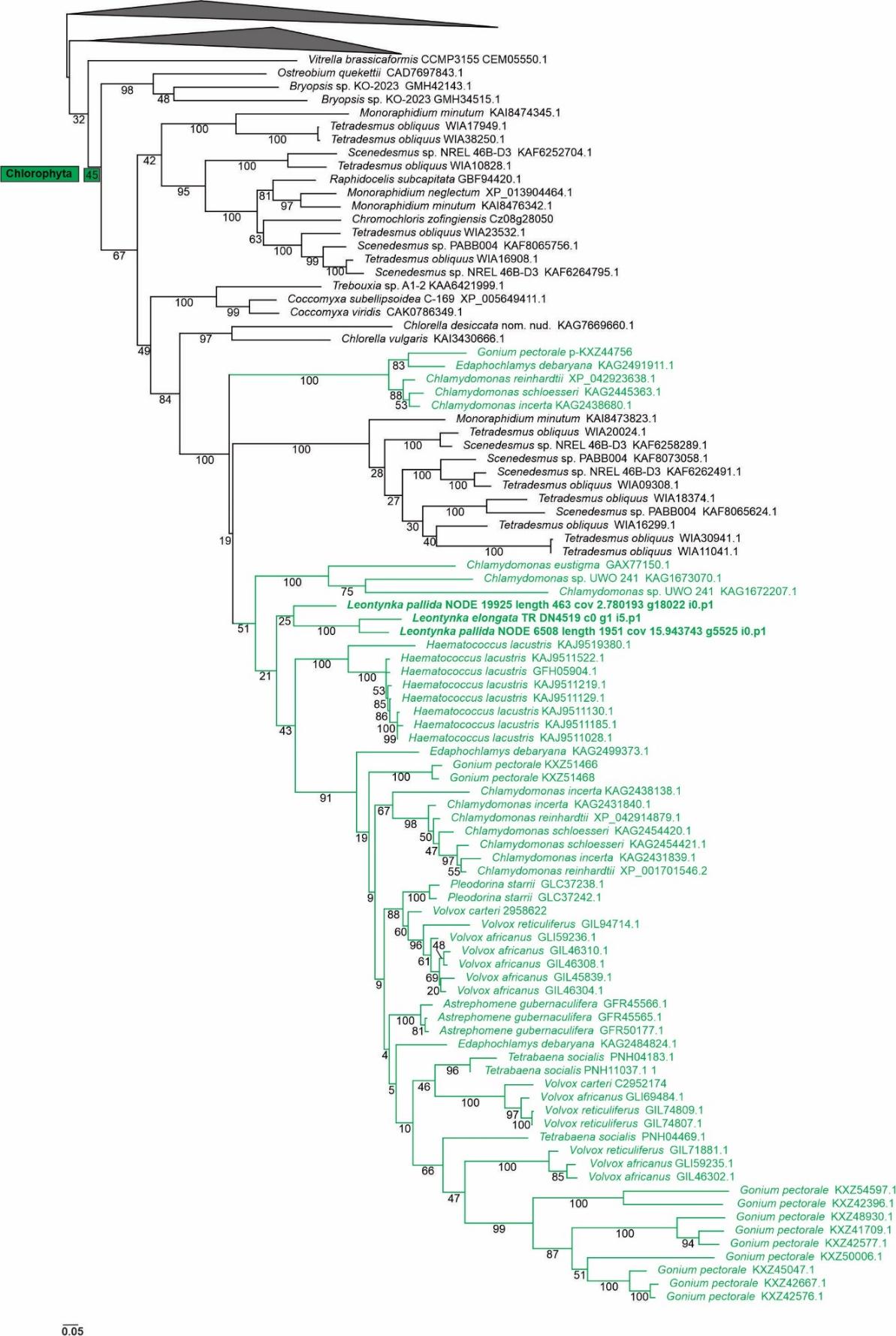
**

**Fig. S6.** **Phylogenetic tree of endo/exo-cellulases.** The tree was computed using IQ-TREE (LG4X model; 100 real bootstraps) and visualized using ITOL. The dataset contained 1,464 protein sequences and 385 positions after trimming. Clades containing outgroup sequences were collapsed as the two triangles on the top. Members of Chlamydomonadales are marked in green.


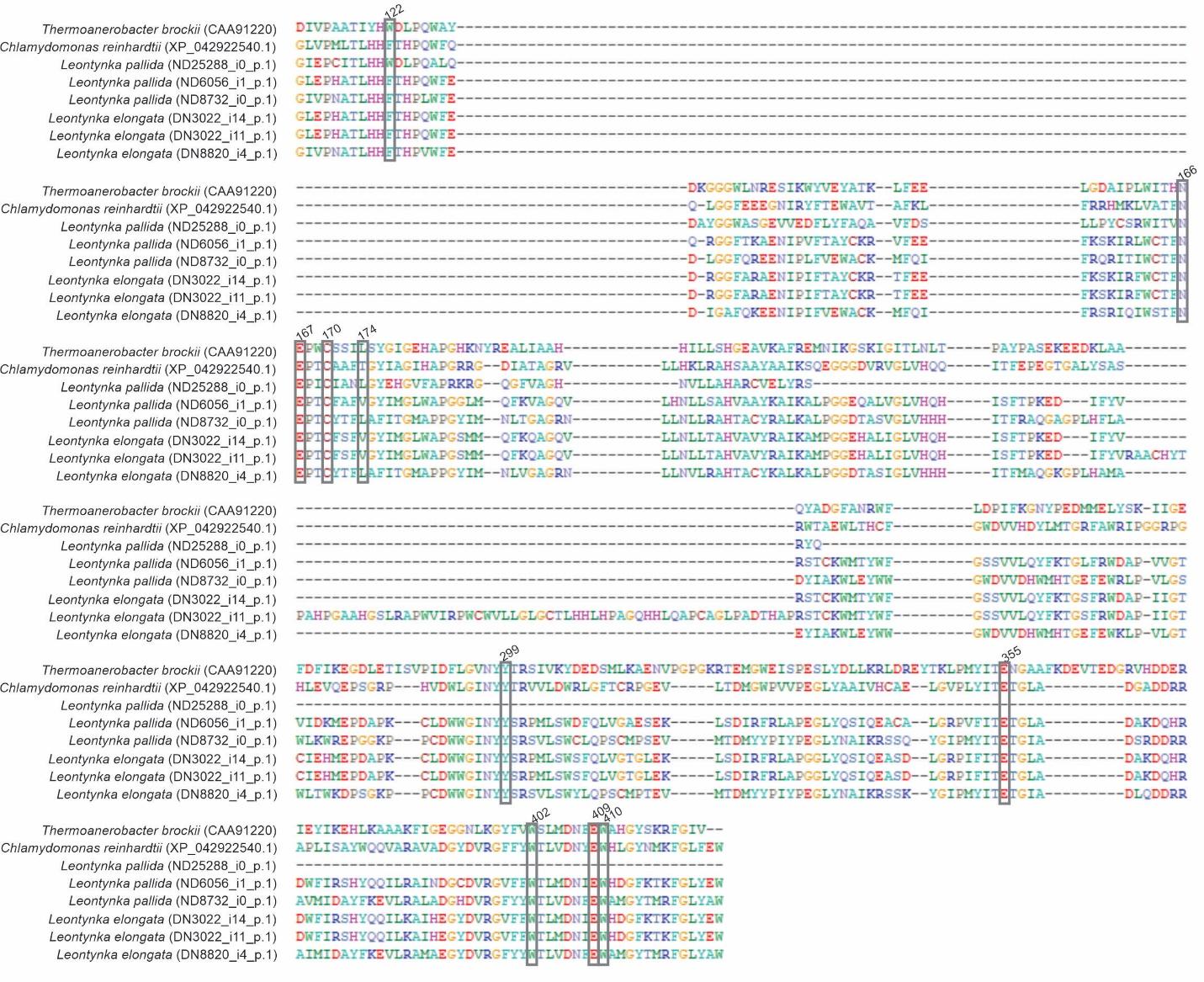


**Fig. S7.** **Multiple sequence alignment of β-glucosidase.** The amino acids responsible for the binding of cellobiose are marked with gray boxes. The coordinates of the critical positions are indicated based on the sequence of the previously studied protein from *Thermoanaerobacter brockii*.


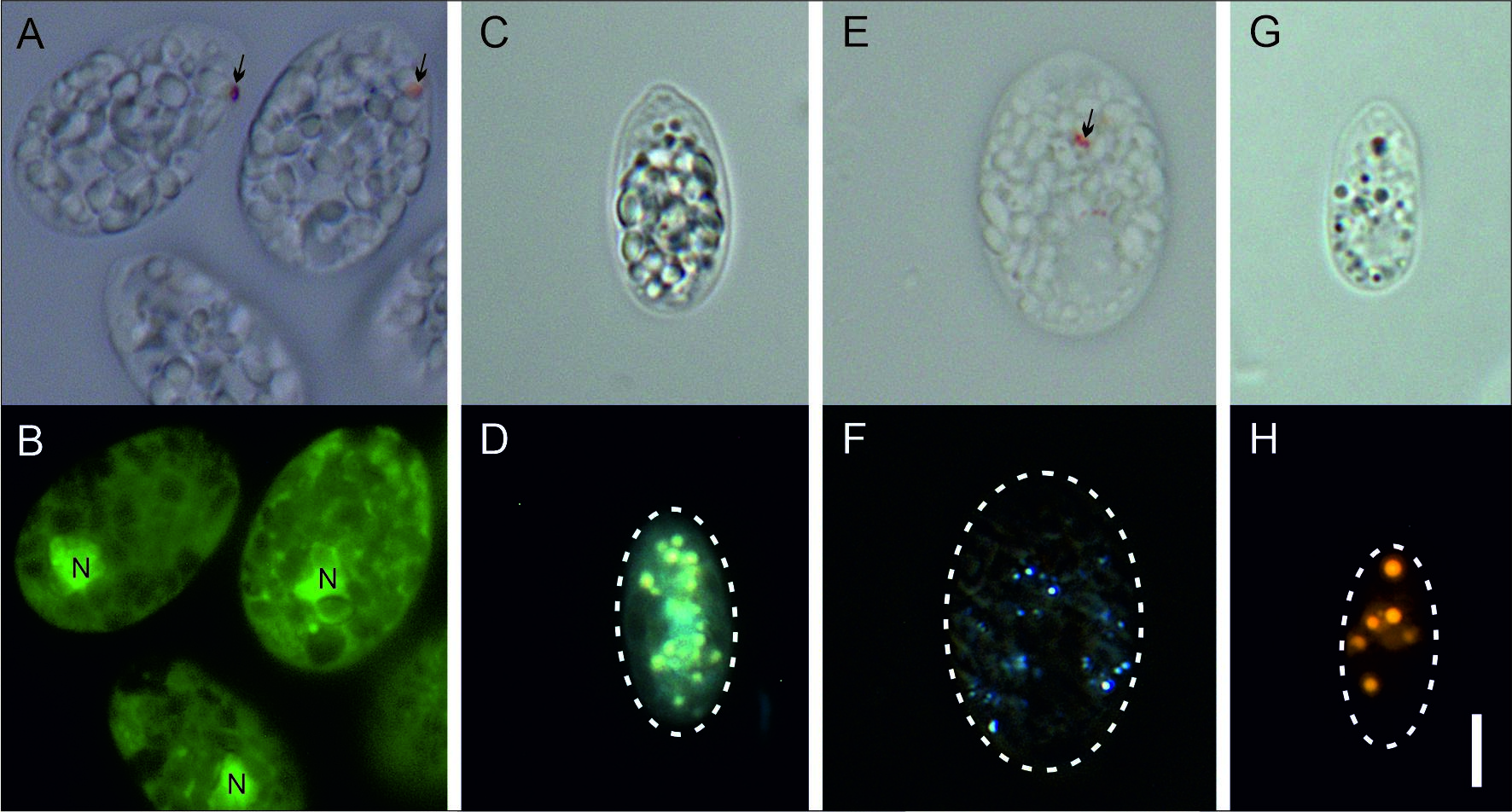


**Fig. S8. Components of *Leontynka elongata* cells visualized by a wide range of staining techniques and polarization microscopy.** Bright field microscopy (A, C, E, G) often reveals numerous starch grains (A, C, E) and the orange eyespot (A, E). SYTO 13 (B) stains DNA and RNA of the nucleus, mitochondria, and plastids. DAPI staining (D) shows polyphosphate granules in yellow and the nucleus in blue. Polarization microscopy (F) shows slightly polarizing starch grains and strongly polarizing birefringent guanine crystals. Nile red staining (H) visualizes lipid droplets. Arrows indicate eyespots, N - nucleus. Scalebar: 5 µm.

**
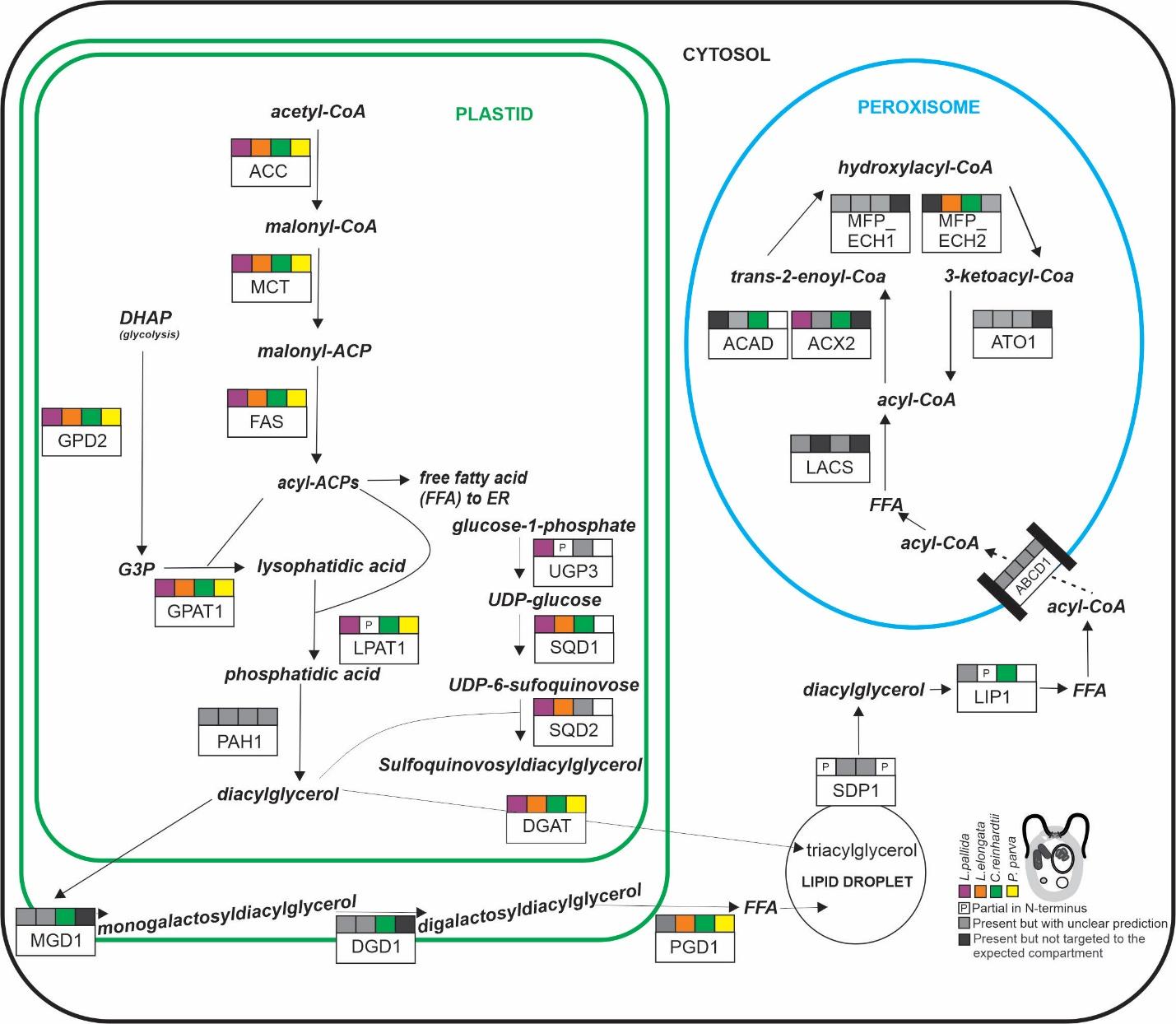
**

**Fig. S9**. **Fatty acid synthesis and degradation in *Leontynka* and its comparison with other chlamydomonadalean algae.** The scheme is based on assessing the presence/absence and (putative) subcellular localization of relevant enzymes for four species, *Leontynka pallida*, *Leontynka elongata*, *Chlamydomonas reinhardtii*, and *Polytomella parva* (see the graphical legend in the bottom right corner). Squares filled with color indicate that our *in silico* localization predictions are consistent with the depicted localization, either as expected or experimentally verified in *C. reinhardtii*. An empty square indicates that the protein was not identified in a given species, and a gray square indicates that the protein was predicted to be localized in a different cell compartment. Abbreviations: acetyl-CoA biotin carboxyl carrier protein (ACC); malonyl-CoA:ACP transacylase (MCT); fatty acid biosynthesis enzymes (FAS; note this stands for multiple different enzymes catalyzing the different steps of the cyclical fatty acyl extension by a two-carbon unit in each cycle); 1-acyl-sn-glycerol-3-phosphate acyltransferase (LPAT1); glycerol-3-phosphate acyltransferase (GPAT1); glycerol-3-phosphate dehydrogenase (GPD2); phosphatidate phosphatase (PAH1); diacylglycerol acyltransferase (DGAT); galactolipid lipase (PGD1); monogalactosyldiacylglycerol synthase (MGD1); UDP-glucose pyrophosphorylase (UGP3); UDP-sulfoquinovose synthase (SQD1); sulfoquinovosyltransferase (SQD2); digalactosyldiacylglycerol synthase (DGD1); patatin-like TAG lipase (SDP1); likely a DAG lipase (LIP1); acyl-CoA synthetase (ACAS); AMP dependent synthetase/ligase (LACS); acyl-CoA dehydrogenase (ACAD); acyl-CoA oxidase (ACX2); 3-hydroxyacyl-CoA dehydrogenase/enoyl-CoA hydratase (MFP_ECH1; MFP_ECH2); acetyl-CoA acyltransferase (ATO1); long-chain acyl-CoA transporter (ABCD1). Source data is deposited in the Additional file 2: Tables **S8**–**S11**.

**Fig. S10. HPLC analyses of pigment extracts from a *L. pallida* culture including the bacterial microbiome, from a sample of the same culture strongly enriched in bacteria, and from freshly prepared ATCC medium.** The upper chromatogram is identical to the respective chromatogram in Fig. 4; asterisks denote the trans-isomer of the respective carotene species. The three main peaks in the bacterial sample eluting between 8 and 11.5 min had absorbance spectra similar to zeaxanthin. The absorbance spectra of the peaks in the chromatogram from the ATCC extract were not typical for carotenoids, showing a relatively sharp absorbance band in the blue region with a maximum at about 435 nm and a shape similar to the Soret band of chlorophylls. The ATCC extract was analyzed to exclude the possibility that some of the carotenoids detected in the extracts from the *Leontynka* cultures were derived from carotenoids already present in the algal culture medium.

**
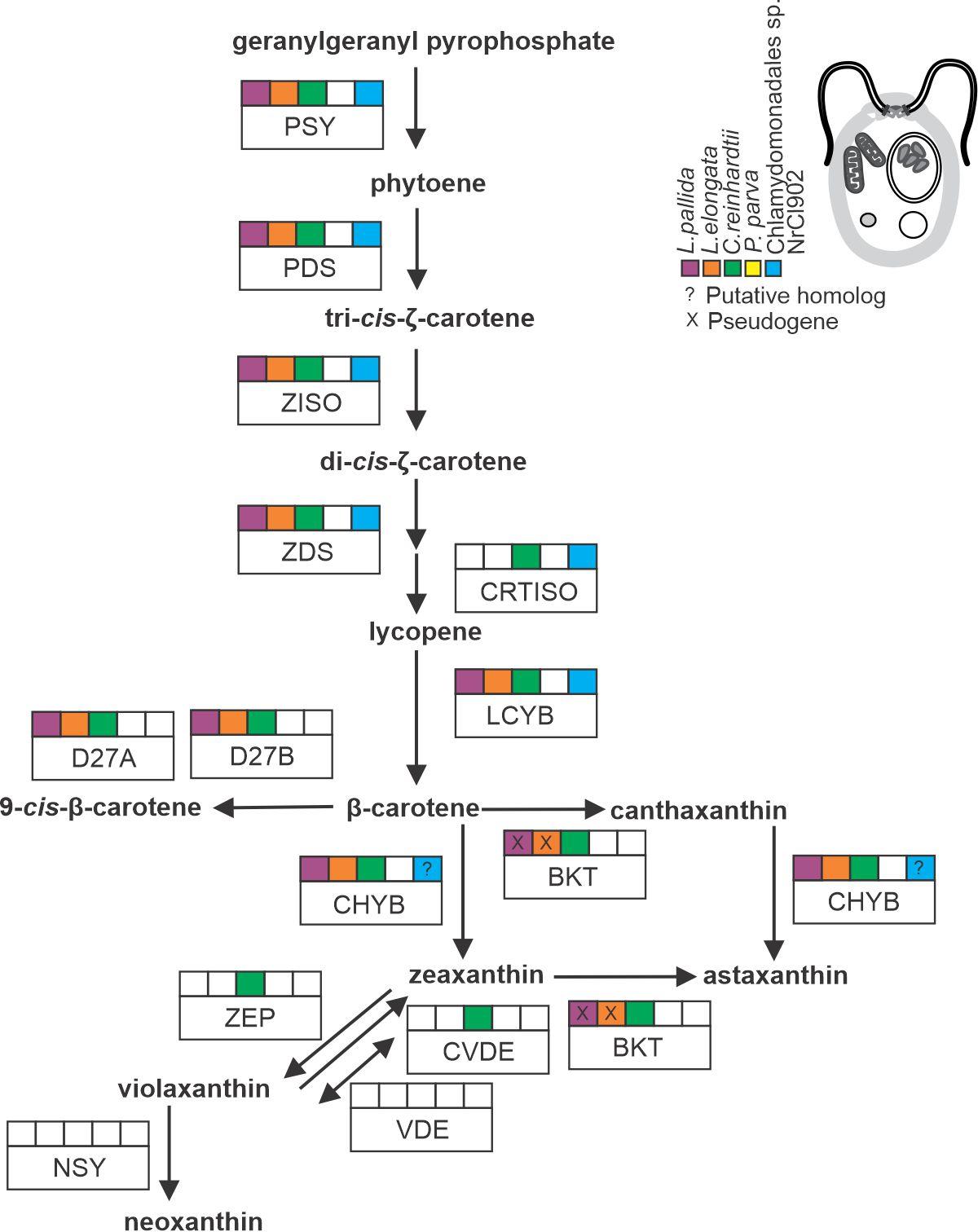
**

**Fig. S11**. **Carotenoid synthesis in *Leontynka* and its comparison with other chlamydomonadalean algae.** The scheme displays the presence/absence and (putative) subcellular localization of relevant enzymes for the same four species as Fig. S7, following the same display conventions (see also the graphical legend in the top right corner). Abbreviations: phytoene synthase (PSY); phytoene desaturase (PDS); ζ-Carotene isomerase (ZISO); zeta-carotene desaturase (ZDS); carotenoid isomerase (CRTISO); lycopene beta-cyclase (LCYB); β-carotene isomerases (D27A; D27B); carotene beta-ketolase (BKT); carotene beta-hydroxylase (CHYB); zeaxanthin epoxidase (ZEP); violaxanthin de-epoxidase (VDE), and neoxanthin synthase (NSY). Source data deposited in Additional file 2: Tables **S8**-**S11**, and **S17**.

**
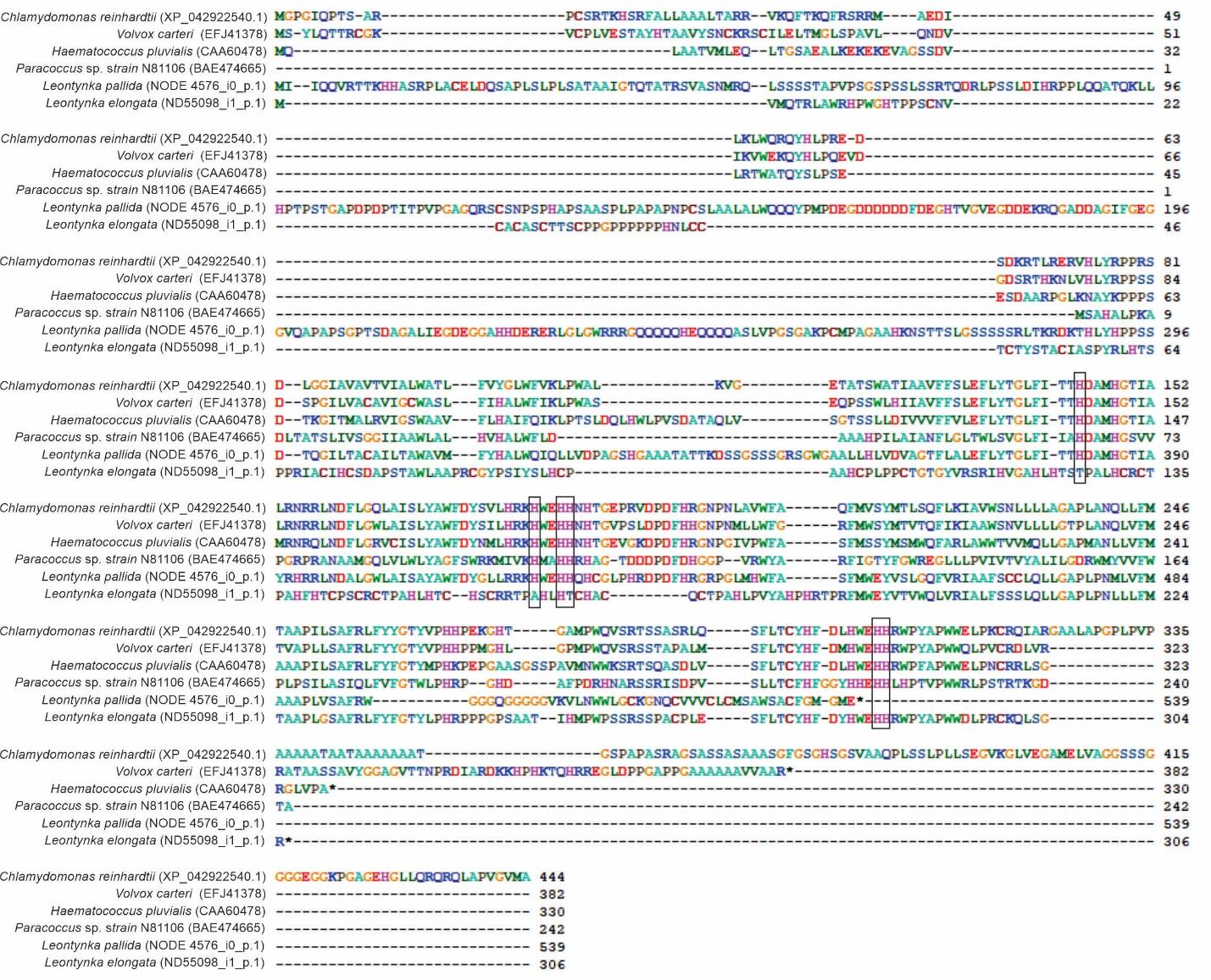
**

**Fig. S12**. **Multiple sequence alignment of β-carotene ketolase (BKT) from green algae.** BKT is involved in carotenoid synthesis and possesses three iron-binding motifs, each of which contains histidine residues essential for catalytic activity [64]. The transcript from *L. pallida*, however, encodes a protein that lacks amino acids at the C-terminus, including the third iron-binding motif. The protein encoded by the transcript from *L. elongata* lacks the N-terminal part containing the first and second iron-binding motif. Fe^2-^ is involved in the catalytic reaction of BKT. The Fe^2-^ binding regions containing conserved histidine residues (and crucial for the function of BKT) are highlighted in boxes. Both transcripts from the *Leontynka* (meta)transcriptome were partial in the assembly and were extended manually from transcriptomic reads.


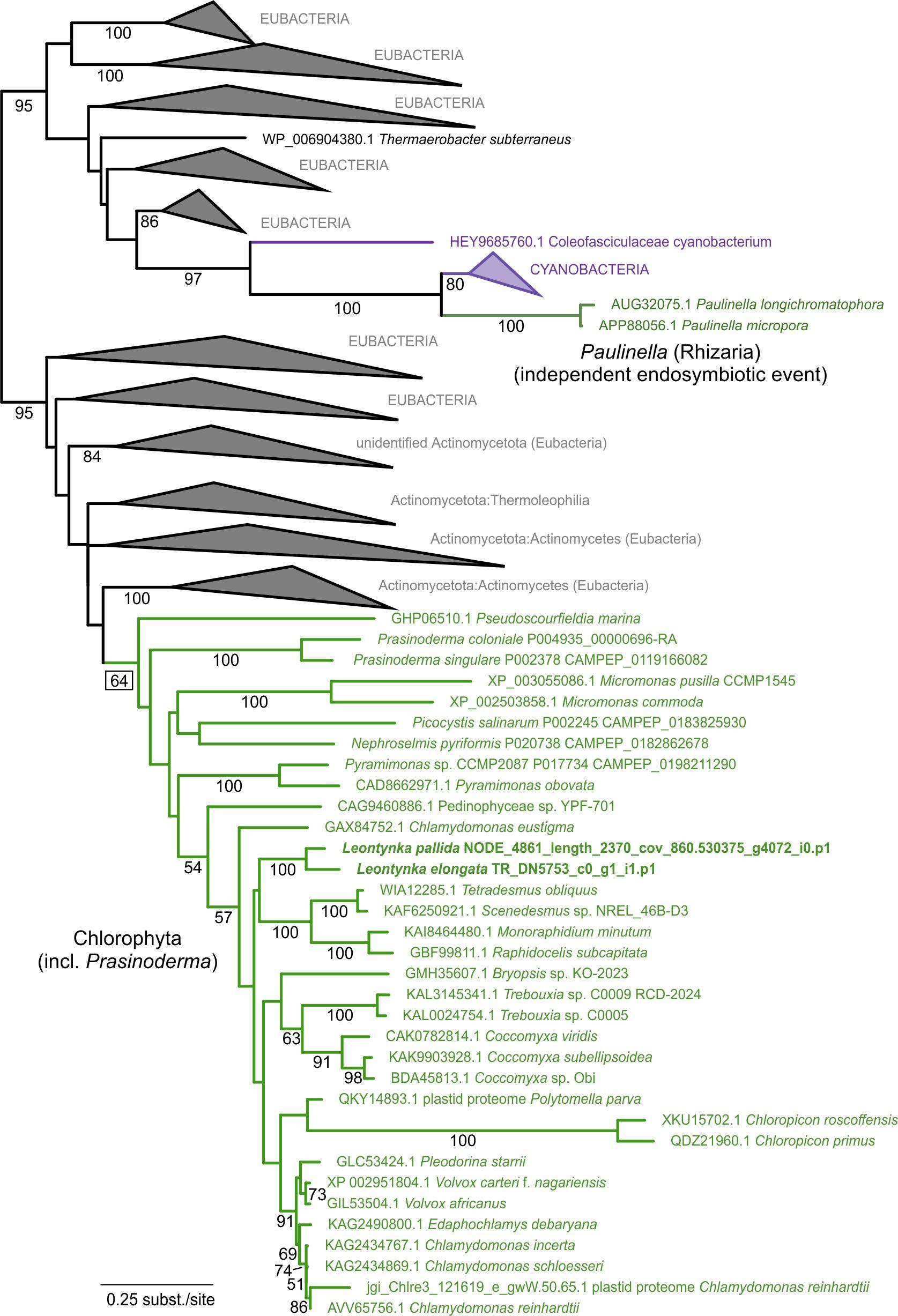


**Fig. S13. Phylogenetic tree of fructose-1,6-bisphosphatase class 2 (GlpX).** The tree was computed using IQ-TREE (LG4X model; 100 real bootstraps) and visualized using ITOL. The dataset contained 307 protein sequences; 317 positions after trimming. Clades containing outgroup sequences were collapsed into triangles. Eukaryotes (all with plastid organelle) are marked in green.


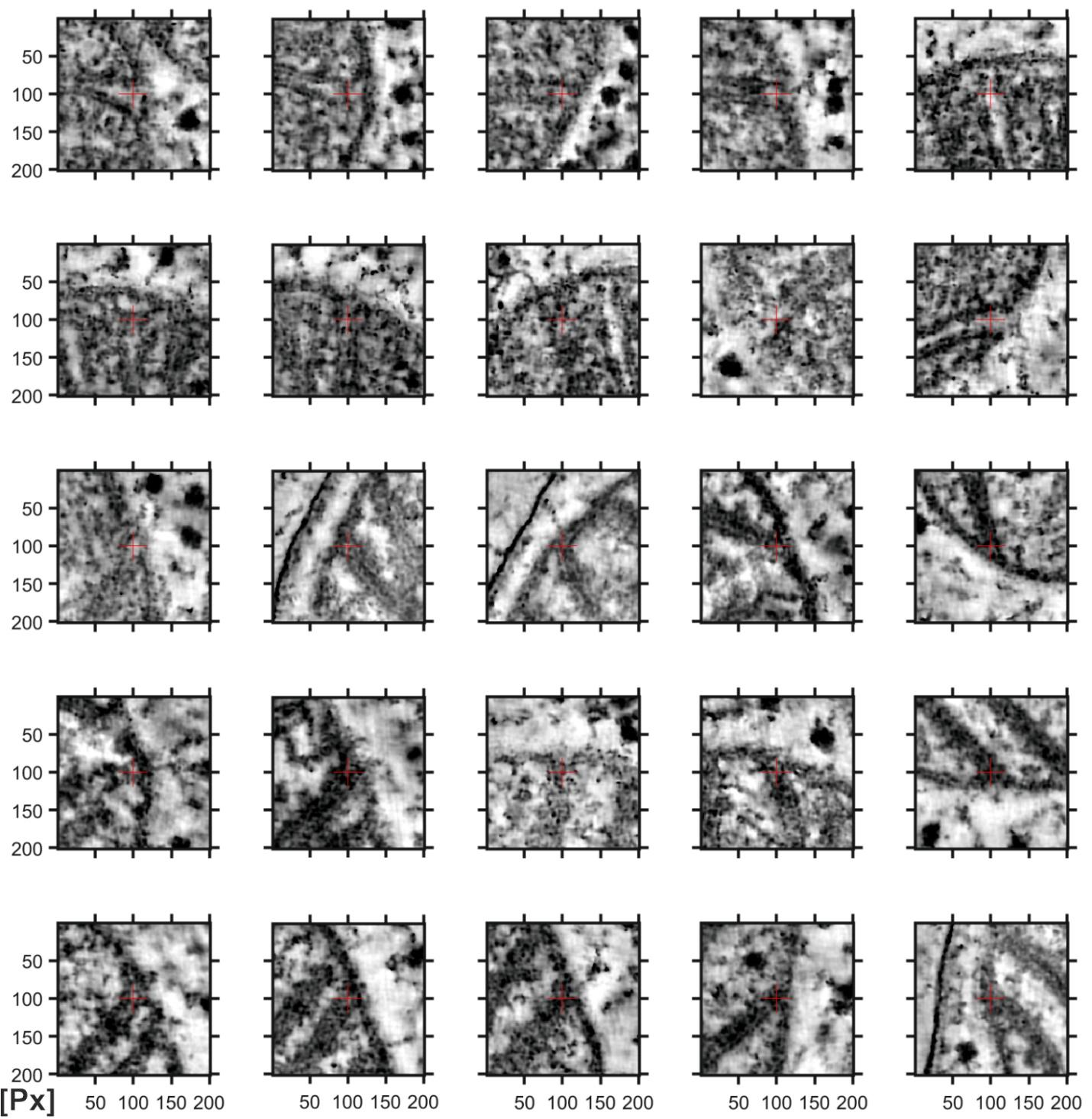


**Fig. S14. Cutouts from tomograms showing all 25 analyzed cristae of *Leontynka elongata* mitochondria in detail with cristae junctions at the center (indicated by a red cross).** Isotropic pixel size is 0.67 nm.


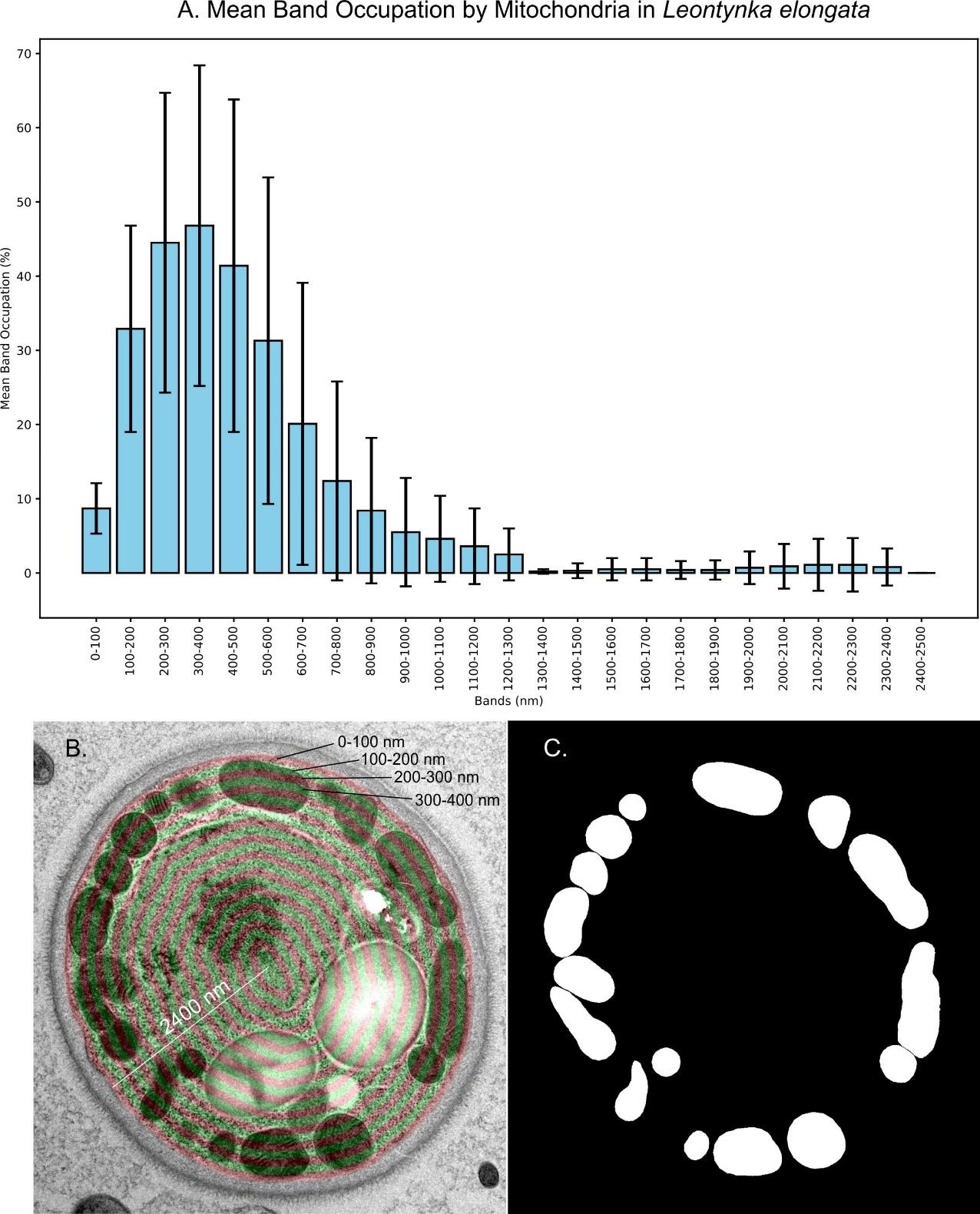


**Fig. S15. Position of mitochondria in *Leontynka elongata* cells. A.** The average percentage of the band area occupied by mitochondria, for each of the 24 concentric bands. Data were obtained using serial section electron tomography (see main text for details). The average cell diameter (cytoplasm, chlamys excluded) was 6077 ± 1164 nm (n=11). **B.** Example of cell cross section with depicted 24 bands, each 100 nm in width. **C.** Corresponding model of mitochondria to the cell shown in panel B. Source data are available as Additional file 2: Table **S12** and **S13**.


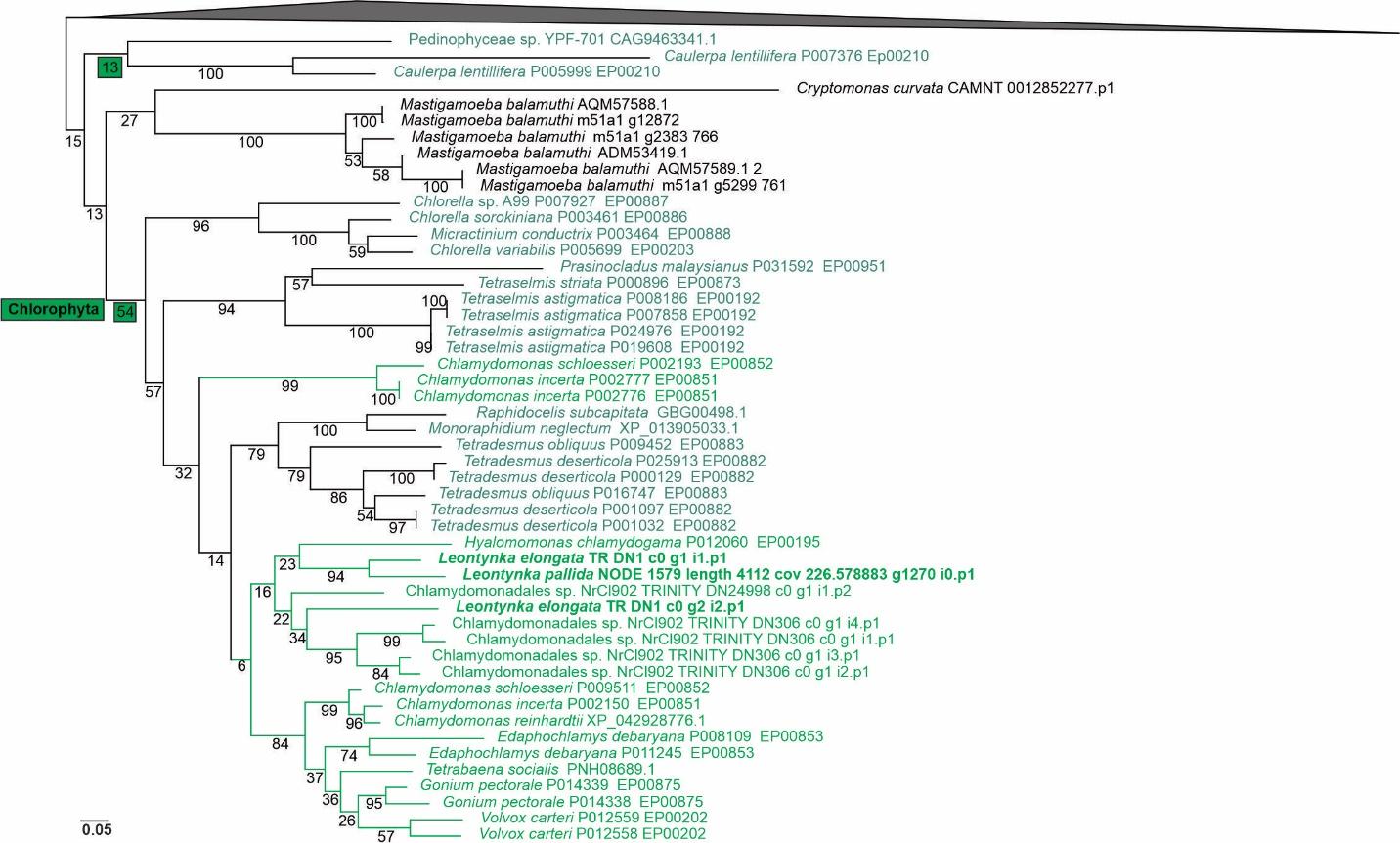


**Fig. S16**. **Global phylogenetic tree of pyruvate formate lyase (PFL).** The tree was computed using IQ-tree (LG4X model; 100 real bootstraps) and visualized using ITOL. The dataset contained 288 protein sequences and 857 positions after trimming. Clades containing outgroup sequences were collapsed as the triangle on the top. Members of Chlamydomonadales are marked in green, other chlorophytes in blue green. This analysis contains complete as well as partial sequences (in contrast to the analysis presented below (Fig. **S18**).

**
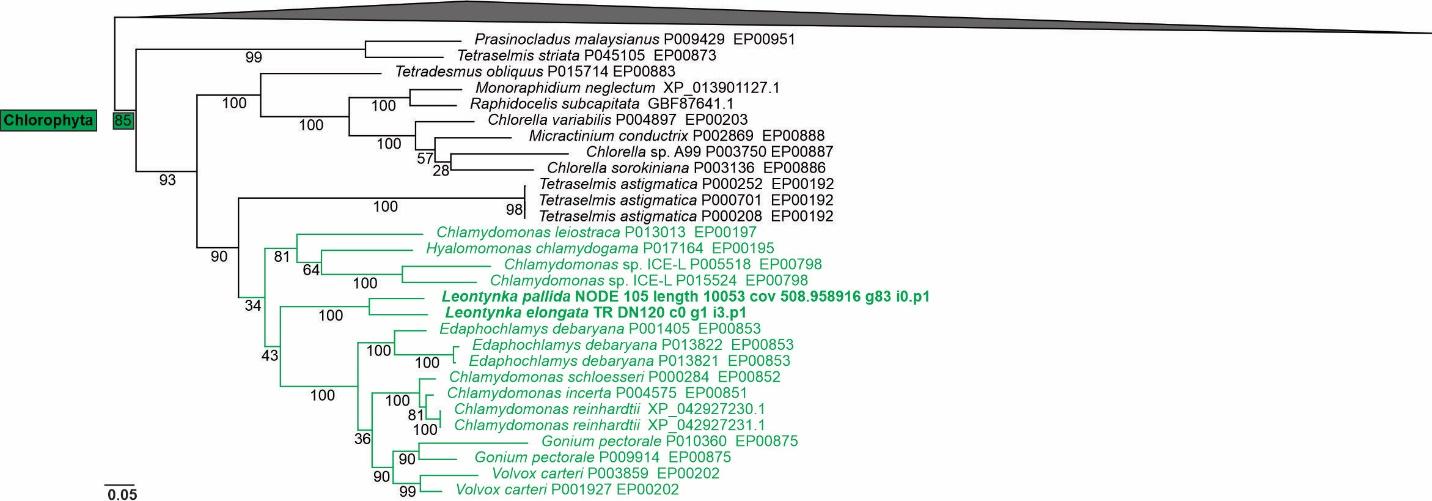
**

**Fig. S17.** **Phylogenetic tree of pyruvate:ferredoxin oxidoreductase (PFO).** The tree was computed using IQ-TREE (LG4X model; 100 real bootstraps) and visualized using ITOL. The dataset contained 716 protein sequences; 1,163 positions after trimming. Clades containing outgroup sequences were collapsed as the triangle on the top. Members of Chlamydomonadales are marked in green.

**
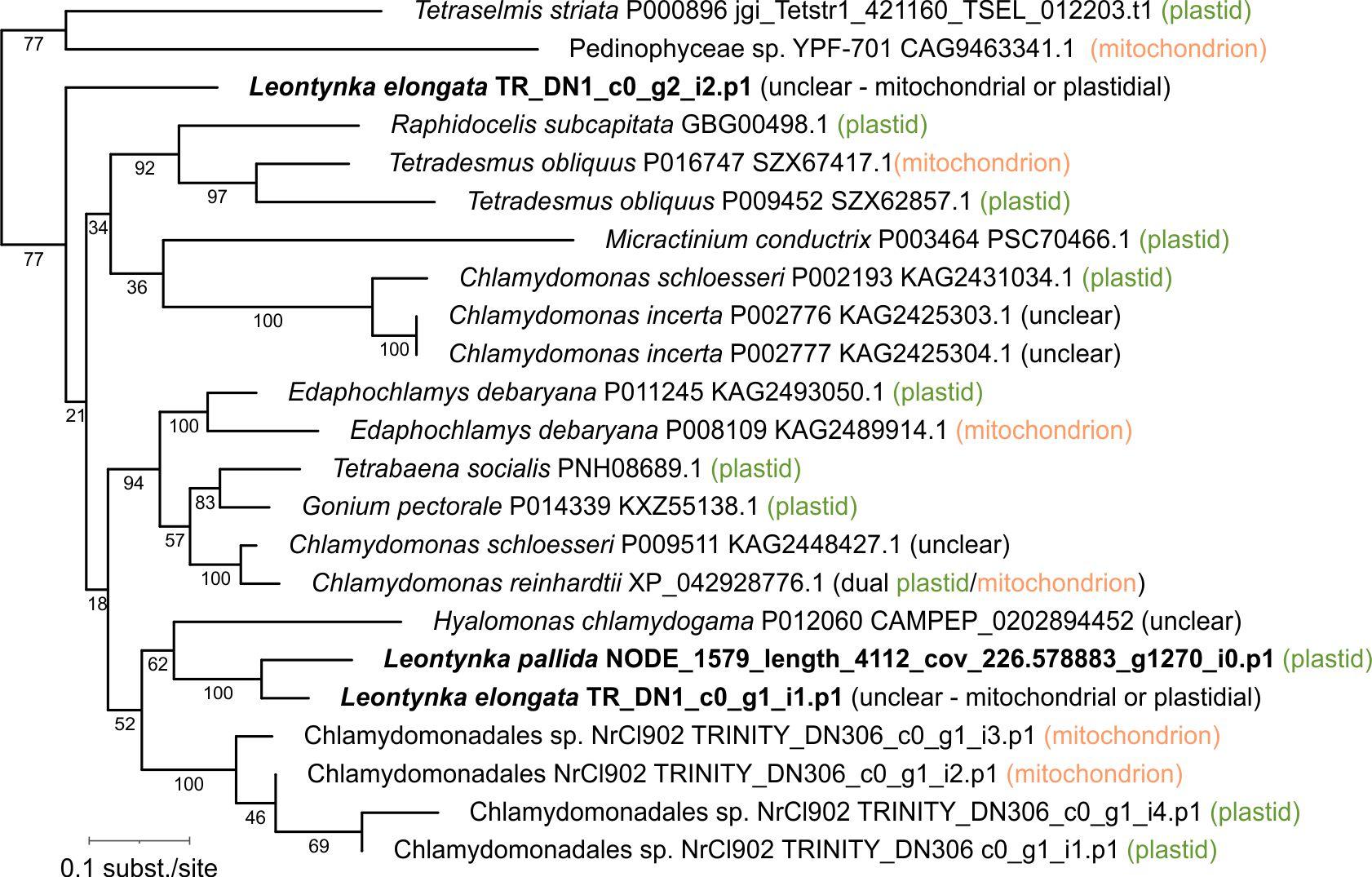
**

**Fig. S18. Focused phylogenetic analysis of pyruvate formate lyase (PFL).** The tree was computed using IQ-TREE (LG4X model with 100 real bootstraps). The dataset contained 23 amino acid sequences and 769 amino acid residues after trimming. Predicted subcellular localization is indicated in the brackets. In *C. reinhardtii*, dual localization has been shown experimentally. Only complete sequences were used in the analysis.


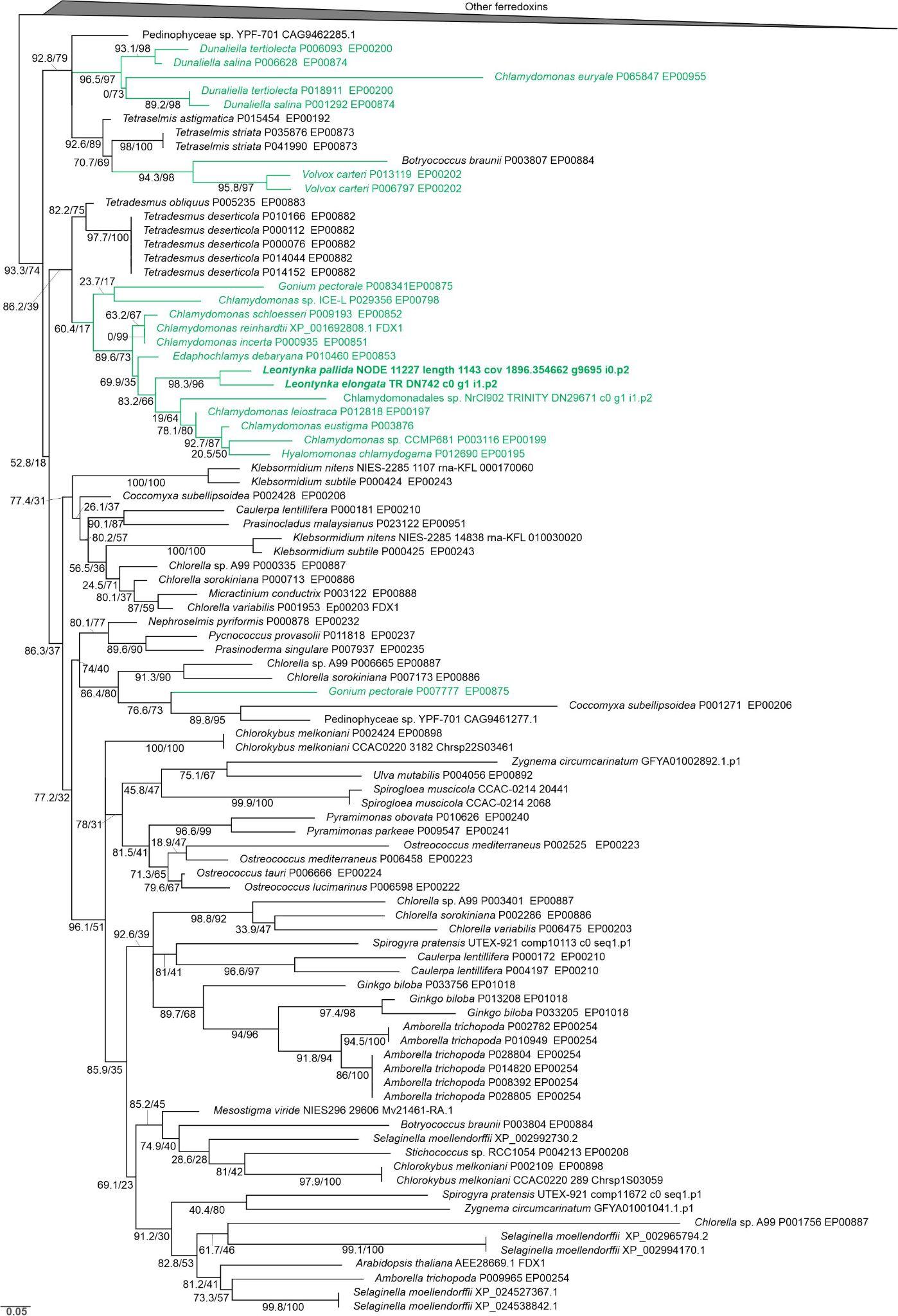


**Fig. S19.** **Phylogenetic tree of ferredoxins from Chloroplastida.** The tree was computed using IQ-TREE (LG+C60+F+G4 model; SH-like approximate likelihood ratio test (SH-aLRT)/1000 ultrafast bootstraps) and visualized using ITOL. The dataset contained 1,047 protein sequences and 98 positions after trimming. The uncollapsed subtree represents ferredoxin 1, the subtree corresponding to other ferredoxin types was collapsed as the triangle on the top. Members of Chlamydomonadales are marked in green.

**
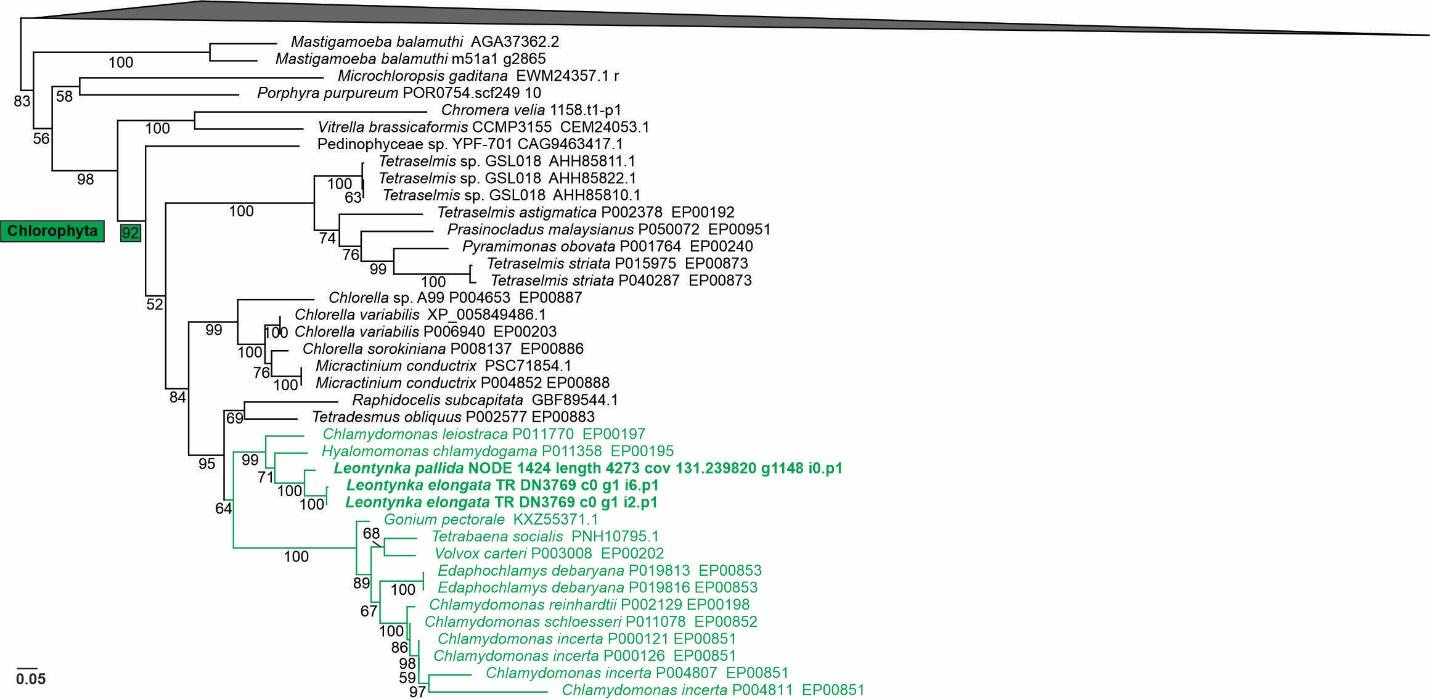
**

**Fig. S20. Phylogenetic tree of the fused hydrogenase maturase protein HydEF.** The tree was computed using IQ-TREE (LG4X model; 100 real bootstraps) and visualized using ITOL. The dataset contained 197 protein sequences and 368 positions after trimming. Clades containing outgroup sequences were collapsed as the triangle on the top. Members of Chlamydomonadales are marked in green.

**
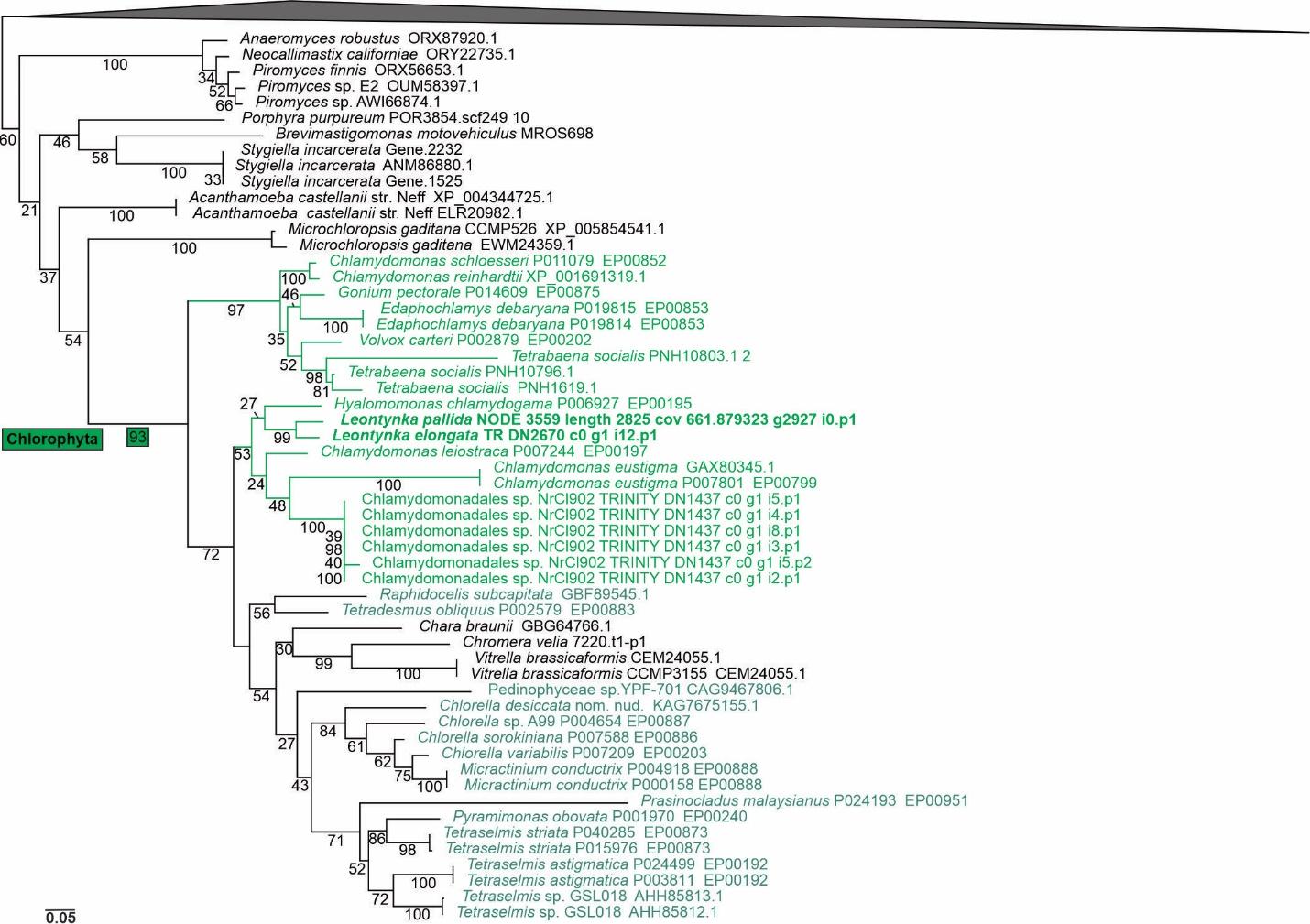
**

**Fig. S21. Phylogenetic tree of the hydrogenase maturase protein HydG.** The tree was computed using IQ-TREE (LG4X model; 100 real bootstraps) and was visualized using ITOL. The dataset contained 341 protein sequences and 1,604 positions after trimming. Clades containing outgroup sequences were collapsed as the triangle on the top. Members of Chlamydomonadales are marked in green, other chlorophytes in blue green.

**
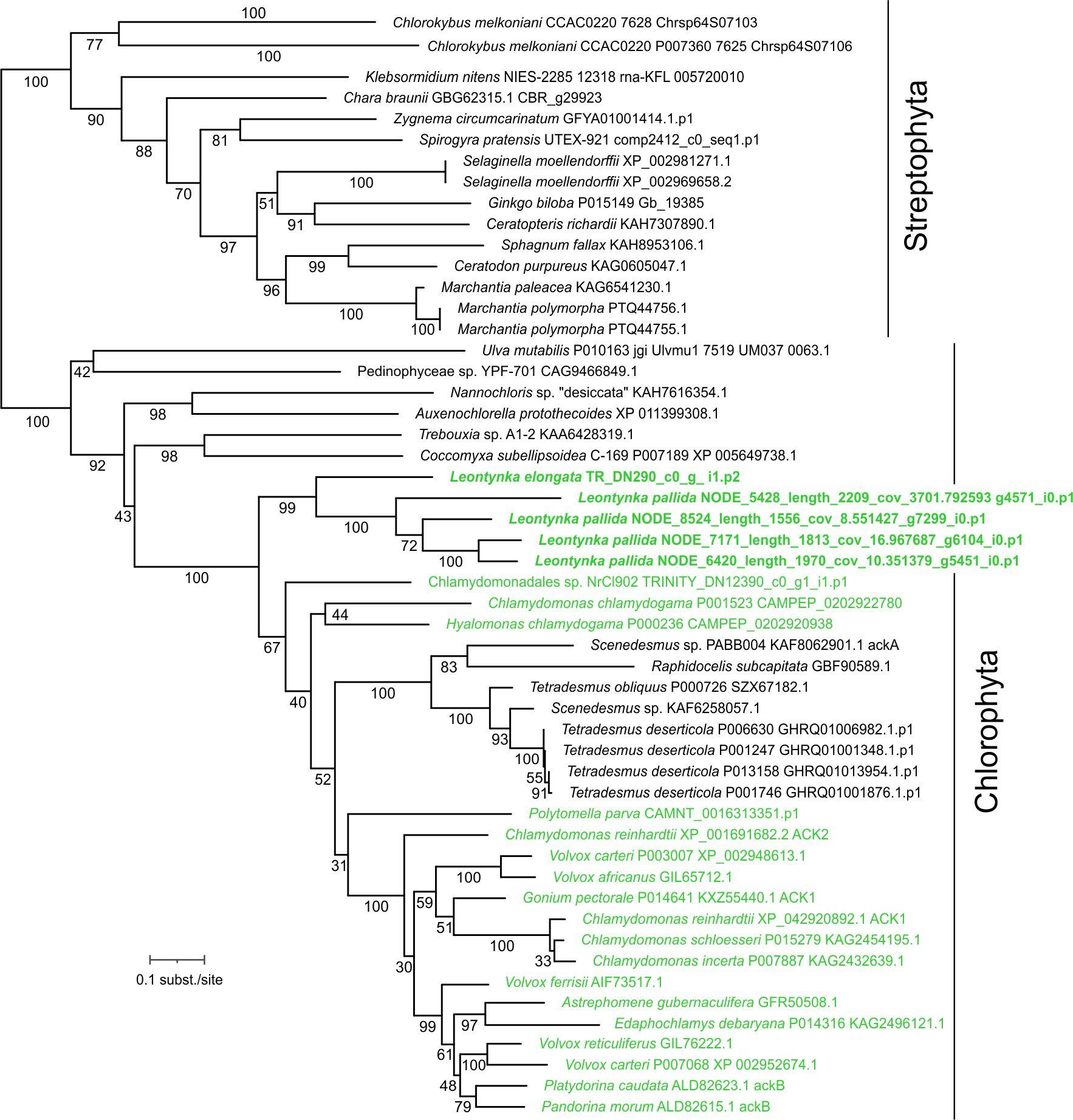
**

**Fig. S22.** **Focused phylogenetic analysis of acetate kinase (ACK).** The tree was generated using IQ-TREE (LG4X model with 100 real bootstraps). The dataset contained 55 amino acid sequences and 396 amino acid residues after trimming. ACK sequences from Chlamydomonadales members are in green. Only complete sequences were used in the analysis. A global phylogenetic analysis of ACK with all identified sequences is available on Figshare.

**
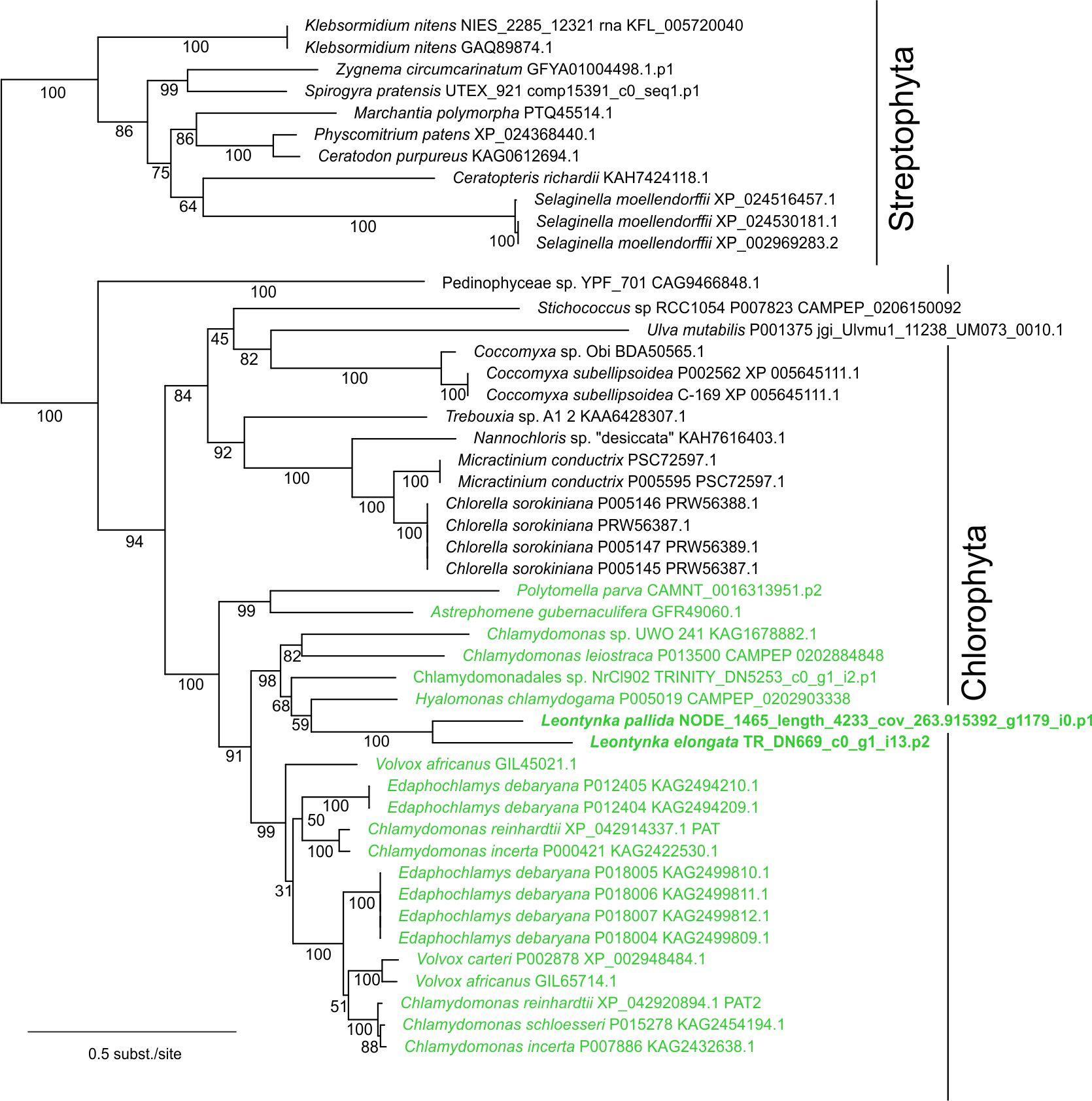
**

**Fig. S23. Focused phylogenetic analysis of phosphate acetyltransferase (PAT).** The tree was computed using IQ-TREE (LG4X model with 100 real bootstraps) and visualized using ITOL. The dataset contained 48 amino acid sequences and 684 amino acid residues after trimming. PATs of the group Chlamydomonadales are in green. The analysis contains only complete sequences. A global analysis of PAT with all identified sequences is available on Figshare.

**REFERENCES**

(numbering based on name text)

64. Ye RW, Stead KJ, Yao H, He H. Mutational and Functional Analysis of the β-Carotene Ketolase Involved in the Production of Canthaxanthin and Astaxanthin. Appl Environ Microbiol. 2006;72:5829–37.
