# Supplementary material for: Energy metabolism and adaptation to hypoxia in the non-photosynthetic green alga Leontynka
Source: BMC Biol. 2026 Jan 29;24:50. doi: 10.1186/s12915-026-02529-3 (PMC12924338; doi:10.1186/s12915-026-02529-3)
Supplement: Supplementary file 3 — Additional file 3: Methods S1 Details of growth experiments. Methods S2 Raman microscopy (settings and image processing). Notes S1 Evaluation of transcriptome completeness. Notes S2 Evaluation of protein localization predictions. Notes S3 Cellulose degradation pathway. Notes S4 Cristae and cristae junctions of Leontynka elongata. [file 12915_2026_2529_MOESM3_ESM.docx]

**ADDITIONAL FILE 3: SUPPLEMENTARY TEXT**

**Article title:** Energy metabolism and adaptation to hypoxia in the non-photosynthetic green alga *Leontynka*

**Authors:** Pia Corre, Jana Pilátová, Tomáš Bílý, Eliška Zadrobílková, Ivan Čepička, Marie Vancová, Martin Lohr, Oliver D. Caspari, Marek Eliáš, Tomáš Pánek

**Methods S1** Details of growth experiments.

**Methods S2** Raman microscopy (settings and image processing).

**Notes S1** Evaluation of transcriptome completeness.

**Notes S2** Evaluation of protein localization predictions.

**Notes S3** Cellulose degradation pathway.

**Notes S4** Cristae and cristae junctions of *Leontynka elongata.*

**Methods S1 – details of growth experiments**

In all experiments, resazurin dye was added to the culture to monitor redox/oxygen conditions (see Additional file 1: Fig. **S1**). Approximately 1,400 *L. pallida* cells were inoculated to the fresh culture medium to 10 ml of the final volume for microoxic and anoxic conditions (maintained in microoxic and anaerobic chamber) and to 5 ml for oxic conditions. The tubes with 5 ml of the medium were left open to secure normoxia in the whole volume. A microoxic chamber (8–9% oxygen) was prepared using a CampyGEN gas generator (Oxoid, Hampshire, UK) placed into an AnaeroJar (Oxoid, Hampshire, UK); an anaerobic chamber (<1% oxygen) was prepared using an AnaeroGEN gas generator (Oxoid, Hampshire, UK). Cell growth in each tested condition (oxygen concentration and growth medium) was analyzed in triplicates. Two different culture media were used: ATCC 802 medium (control) and ATCC 802 medium with added acetate (17 mM). Cultures were monitored every 72 hours for 15 days. All cultures were maintained in darkness at room temperature. All replicates were subjected to manual cell counting. Cells were stained using 0.2% trypan blue to distinguish viable and non-viable cells. Counting was done in a Bürker-Türk counting chamber (Paul Marienfeld, Germany) under an Olympus BX51 microscope. To observe starch granules in the cells, the cultures were kept in an AnaeroJar for 8 days under anoxic and microoxic conditions with AnaeroGEN and CampyGEN, respectively.

**Methods S2 – Raman microscopy (settings and image processing)**

We used grating of 600 g/mm, a UHTS300S detector with the spectrograph centered at 2,250 cm^- 1^ providing a wide range from the fingerprint region to high-wavenumbers, *i.e*., 220–3,850 cm^-1^. Spatial resolution for imaging has been set closely below the diffraction limit to 200 nm in x-y dimensions with the high confocality obtaining 1µm thick optical sections. The green 532nm excitation laser was set to the output of ~20 mW at the focal plane of the sample and the 0.1s integration time. We optimized the measurement process be able to correctly measure carotenoids, namely we had to (1) depict the unaltered carotenoid localization with a low laser power; (2) to apply photobleaching with a high laser power; and (3) to reveal the chemical composition of other cellular structures. In order to depict carotenoid distribution not affected by photooxidative changes over the intense laser exposure, the power was decreased by 10-500× to 50 µW. The optimal setup to depict the wide dynamic range of the strong carotenoid signal in lipid droplets and the considerably weaker but well-detectable signal in the plastid membranes was around 2.5 mW. We measured at least 5 cells per cultivation condition per species in biological replicates with both low and high laser intensity output, *i.e.*, 2.5 and 25 mW. Collected data were pre-processed for cosmic rays and background subtraction with spectral cropping using the WITec Project 6.0 software. Subsequently, the multidimensional spectral data was decomposed into separate chemical images and their respective spectral components were plotted in SigmaPlot 12.5.

**Notes S1 – Evaluation of transcriptome completeness**

The completeness of *L. pallida* and *L. elongata* proteomes predicted from transcriptome assemblies was assessed using BUSCO v.5 and the chlorophyta_odb10 dataset, which contains 1,519 single-copy orthologs. The percentage of missing genes in *Leontynka* spp. was relatively low (see Additional file 1: Fig. **S2A**), at 11.5% for *L. pallida* and 15.7% for *L. elongata*. This is an even better score than predicted proteome from the *Polytomella parva* reference transcriptome (21.1%), while the latest predicted proteome of *C. reinhardtii* (v6.1) displays only 0.1% missing data in the BUSCO analysis. However, it is important to consider that *Leontynka* and *Polytomella* lost many genes connected to photosynthesis. To mitigate the effect of these genes in the BUSCO analysis, we performed another analysis using the eukaryote_odb10 database, which contains 255 protein-coding genes present in both algae and heterotrophic eukaryotes (see Additional file 1: Fig. **S2B**). *C. reinhardtii* was missing 3.9% of the proteins, *L. pallida* was missing 10.6%, and *L. elongata* was missing 15.3% of proteins from this dataset. Based on this analysis, we consider the *Leontynka* data (especially from *L. pallida*) to be high-quality transcriptomes with approximately 11% and 15% missing genes in respective transcriptome assemblies. Regardless, the absence of particular genes in *P. parva* and *Leontynka* spp. should be interpreted with caution as these datasets are transcriptome assemblies, and genes that were not transcribed at the time of RNA extraction are lacking in the data while they are present in the nuclear genome.

**Notes S2 – Evaluation of protein localization predictions**

Critical to any metabolic reconstruction based on sequence data is the proper consideration of the subcellular localization of the different proteins encoded by the organism. Despite *Leontynka* being a non-model organism that is difficult to study using wet-lab experiments, we took advantage of extensive research on its relative *C. reinhardtii* to make our *in silico* predictions as accurate as possible. Critically, the subcellular localization of many proteins encoded in the *C. reinhardtii* genome has been verified experimentally; either proteomics on isolated organelles [14,15] or by large-scale tagging followed by fluorescence microscopy [16]. This allowed us to select the best-performing tools for *in silico* prediction of protein subcellular localization for Chlamydomonadales representatives by testing predicted against experimentally validated localizations of selected *C. reinhardtii* proteins (see Additional file 2: Tables **S4**–**S7**). One tool, PredAlgo [17], has been designed specifically to predict the intracellular localization of proteins in green algae based on training data from *C.* *reinhardtii*. Indeed, PredAlgo performs very well, but Deeploc2.0 and DeepLoc2.1 [18] predictions were even more accurate in our test (and comparable). We decided to complement the two predictors with TargetP-2.0 [19] but it has to be pointed out that only ~50 % of plastid-targeted proteins from *C. reinhardtii* are recognized as such but TargetP-2.0 and the situation is even worse for mitochondrial-targeted ones (~40 %). We did not use WoLF PSORT [20] for our final localization predictions because it has an extremely high rate of false positives erroneously predicted to the plastid instead of the mitochondrion (>50%). A simple majority consensus between the three selected predictors has been used to assign protein localization with one exception: when a protein was predicted in the category “others” of TargetP-2.0 and PredAlgo, but DeepLoc2.1 assigned it as a cytosolic or peroxisomal, we marked it as such. This is because the former two predictors do not distinguish peroxisomal and cytosolic proteins. We used this conservative approach to avoid false positives. We expect a significant portion of plastid and mitochondrial proteins to stay unassigned. On the other hand, the probability that we assigned a protein as mitochondrial when it is localized to the plastid (or vice versa) is very low. To double-check the quality of our predictions, we evaluated whether predicted plastid and mitochondrial proteins from *Leontynka* spp. (see Additional file 2: Table **S8**-**S11**) showed typical features of chloroplast transit peptides (cTP) or mitochondrial transit peptides (mTP). Transit peptides vary considerably in sequence, but do rely on critical physico-chemical features. mTPs are described as a positively-charged amphipathic helix starting at the N-terminus. cTPs show a tripartite structure [21] with a less-charged N-terminal stretch [22], a central positively-charged part that is unstructured in solution but may fold into an amphipathic helix in a membrane-environment [23], and a C-terminal part that contains and can stretch beyond a cleavage site, encompassing mainly unstructured sequence. While there is uncertainty about the actual length of cTP parts for any given sequence, for the purposes of this analysis we considered the first 15 residues as the N-terminus, followed by a 20-residue central part and finally a C-terminus of 25 residues. This approach considers the total cTP-length to be 60 residues, matching the length requirements found in *C. reinhardtii* and land plants [24,25]. In our analysis, predicted *Leontynka* cTPs and mTPs show the expected charge distribution (see Additional file 1: Fig. **S3A-D**), with cTPs relatively uncharged at the N-terminus and most positively charged in the central part, and mTPs most positively charged at the very beginning. We checked for Hsp70-binding sites as these may be important for targeting [26], although here we found that all *Leontynka* proteins contain predicted Hsp70-binding sites within the first 60 residues anyway (see Additional file 1: Fig. **S3E**). Semi-conserved ‘FGLK’-sites are thought to play a role in cTPs interacting with the chloroplast import machinery [26]. In *Leontynka* sequences (see Additional file 1: Fig. **S3F-H**), we found a high prevalence of related ‘GLK’-sites in the N-terminal ~40 residues of predicted plastid proteins, as well as in the first ~20 residues of predicted mitochondrial proteins, matching the findings in *C. reinhardtii* [27]. Sentinel amino acids also follow the expected patterns (see Additional file 1: Fig. **S4**): negatively charged residues aspartate and glutamate are almost absent from mTPs and N-terminal/central parts of cTPs. Among positively charged residues, lysines show a reduced prevalence at the N-terminus, with most of the positive charge distribution being attributable to arginines, in line with arginines being more important than lysines for targeting [27]. Finally, cTPs have been characterized to contain phosphorylation sites, and especially have a high prevalence of serines [28]. In *Leontynka*, serines appear more prevalent in the N-termini than in the rest of proteins across all sequences, but we do see serine highest in cTPs and lowest in mTPs in the N-terminal 15 residues. Overall, *Leontynka* proteins predicted to target the plastid or the mitochondrion thus closely match features of cTPs and mTPs generally, and those of *C. reinhardtii* in particular, giving us confidence that our localization predictions are accurate. Indeed, our analysis suggests that we may be underpredicting plastid and mitochondrial localizations. The average across sequences of the ‘Other’ category, which includes proteins that could not be unambiguously assigned a localization, shows some deviation from the baseline expectation that the N-terminus should be identical to the rest of the sequence. For example, the net charge of ‘Other’ N-termini is slightly positive, negatively charged residues are less prevalent, and serines are more prevalent than later on in the sequences. These findings suggest that the set of ‘Other’ proteins likely contains some proteins equipped with cTPs or mTPs. In the trade-off between false positives and false negatives, we, therefore, appear on balance to be eliminating false positives and accepting more false negatives. For the purposes of our current study, this finding gives us confidence that those plastid and mitochondrial localizations we do assign are most likely correct.

**Notes S3 – Cellulose degradation pathway**

The pathway involves several enzymes, namely endo-β-1,4-glucanases, which cleave the cellulose chain at random positions, and exoglucanases, which cleave the cellulose molecule from the cellulose chain end. Cellulose outside the cell is catabolized into cellobiose (CB) and cellodextrin (CD), which are then transported into the cell via unspecific transporters, such as major facilitator superfamily (MFS) or ABC (ATP-binding) transporters [8]. Despite being most probably present in the common ancestor of Chlamydomonadales, cellulases are not omnipresent in the group. The *P. parva* transcriptome assembly lacks transcripts for extracellular cellulases, whereas downstream enzymes required to convert CB and CD into glucose are present. From the two aforementioned transporters, we found MFS in both *Leontynka* species. The whole pathway is reconstructed in Fig. **3**.

**Notes S4 – Cristae and cristae junctions of *Leontynka elongata***

Our study provides data that go behind making *Leontynka elongata* the first confirmed representative of the supergroup Archaeplastida with discoidal cristae. As stated in the main text, previous classical TEM studies reported cristae resembling discoidal morphotype in various green algae. Together with these earlier reports, our findings indicate that discoidal cristae are more widespread in green algae and have been largely overlooked due to the focus on the plastid organelle in these organisms. Because our study presents the first 3D reconstruction of discoidal cristae outside the supergroup Discoba, it also provides valuable comparative data to study cristae structure and evolution in general. In *T. brucei*, the lumina of discoidal cristae are 34.4 ± 11.9 nm in width and 80.0 ± 44.6 nm in length (n=6) [76,77]. We used the same methodology for the sample preparation and revealed that cristae of *L. elongata* share the general characteristics of the discoidal cristae from *T. brucei*, including the size and shape of CJs (tubular with similar diameter). However, the discoidal cristae of *L. elongata* are significantly larger than those of *T. brucei* (116.0 ± 25.7 nm in height; 182.2 ± 71.5 nm in width), being five times wider*.* The CJs of *L. elongata* are circular, with a diameter of 21.1 ± 3.4 nm (n=25) (Fig. **8F**; see Additional file 1: Fig. **S14**). Hence, they are similar to *T. brucei* (25 ± 5.6 nm in diameter) [76,77].

**REFERENCES**

(see the main text for references)
